# Workplace size and cardiovascular disease subtypes among 11 million Korean wage workers: a nationwide cross-sectional study

**DOI:** 10.1186/s12889-026-28385-y

**Published:** 2026-07-08

**Authors:** Yangwoo Kim, Minji Koo, Eun Mi Kim, Jaiyong Kim, Inah Kim

**Affiliations:** 1 Department of Social and Preventive Medicine, Inha University College of Medicine, Incheon, Republic of Korea; 2 Department of Occupational and Environmental Medicine, Inha University Hospital, Incheon, Republic of Korea; 3 Department of Artificial Intelligence, National Health Insurance Service, Wonju, Republic of Korea; 4 Department of Big Data Management, National Health Insurance Service, Wonju, Republic of Korea; 5 Graduate School of Public Health, Hanyang University, Seoul, Republic of Korea; 6 Department of Occupational and Environmental Medicine, Hanyang University College of Medicine, Seoul, Republic of Korea

**Keywords:** Cardiovascular disease, Workplace size, Health inequality, Occupational health

## Abstract

**Background:**

Cardiovascular disease (CVD) encompasses distinct conditions whose risk factor profiles and socioeconomic gradients diverge. Workplace size is an administrative marker related to regulatory protections, health service access, and working conditions, but population-based evidence on whether CVD prevalence differs by subtype across workplace-size categories is limited. We examined the association between workplace size and the prevalence of 10 CVD subtypes among Korean wage workers.

**Methods:**

This nationwide cross-sectional study included 11,212,512 Korean wage workers from the National Health Insurance Service database (2021). CVD cases were identified across 10 ICD-10 subtypes (I00–I99; three or more claims). Workplace size was classified into eight categories aligned with regulatory thresholds under the Korean Occupational Safety and Health Act. Logistic regression estimated adjusted odds ratios (aORs), controlling for sex and age, with the 5–29-worker category as reference. Sensitivity analyses applied hospitalization-based case definitions.

**Results:**

Crude hypertensive disease (I10–I15) prevalence was highest in one-person workplaces (11,273.5 per 100,000), but after age and sex adjustment the crude OR of 1.12 changed direction to an aOR of 0.85 (95% CI 0.83–0.87). Mid-range workplaces (30–999 workers) showed the highest hypertensive disease aORs (up to 1.09). In contrast, ischemic heart disease (IHD; I20–I25) aORs were modestly elevated in the smallest workplaces (one-person: 1.12, 95% CI 1.05–1.19; 2–4-person: 1.05, 1.03–1.08), and cerebrovascular diseases (I60–I69) showed a modestly elevated aOR in the largest workplaces (1000 or more: 1.06, 1.03–1.08). Hospitalization-based sensitivity analyses showed IHD crude ORs remaining elevated and strengthening under more stringent criteria.

**Conclusions:**

The association between workplace size and CVD prevalence was heterogeneous across subtypes. Hypertensive disease showed the highest adjusted prevalence in mid-range workplaces, a pattern consistent with demographic composition differences, whereas IHD and cerebrovascular diseases showed divergent prevalence patterns across workplace sizes. These findings indicate that the relationship between workplace size and CVD prevalence varies by subtype, with no uniform pattern across all workplace-size categories, and support disease-specific approaches to workplace-based cardiovascular surveillance. Because adjustment was limited to age and sex, these associations should be interpreted as descriptive prevalence patterns rather than causal effects.

**Supplementary Information:**

The online version contains supplementary material available at https://doi.org/10.1186/s12889-026-28385-y.

## Background

Cardiovascular disease (CVD) remains the leading cause of death worldwide, with an estimated 18.6 million deaths in 2019 [1]. This burden falls disproportionately on working populations: long working hours contribute substantially to ischemic heart disease and stroke [2], and socioeconomic gradients in CVD incidence and mortality are well documented [3]. In South Korea, cardiovascular diseases including stroke are recognized as compensable occupational diseases, with claims rising markedly after the 1998 economic crisis amid intensified overwork [4]. Understanding how workplace characteristics are associated with cardiovascular health is therefore both a scientific priority and a matter of health equity.

Workplace size is one such characteristic. It is an administrative marker related to regulatory protections, health management infrastructure, and working conditions. In South Korea, the Occupational Safety and Health Act imposes graduated obligations at thresholds of 5, 30, 50, 100, 300, and 1000 workers, creating a regulatory gradient in occupational health service requirements [5]. At the bottom of this gradient, workplaces with fewer than five workers are exempt from most provisions of the Labor Standards Act, including rules on working hours, dismissal, and overtime pay. This regulatory stratification intersects with socioeconomic position: employment in smaller workplaces correlates with lower income and has been associated with higher CVD incidence and mortality in the Korean population [3]. Yet most studies of workplace size and cardiovascular health have examined only hypertension or metabolic syndrome [6, 7], leaving the relationship with diagnosed CVD and its subtypes largely unexplored.

This gap is consequential. CVD is not a single entity but encompasses etiologically distinct conditions — ischemic heart disease, cerebrovascular disease, heart failure — whose risk factor profiles and population gradients diverge substantially [8]. In the Korean population, stroke exhibits an inverse socioeconomic gradient whereas ischemic heart disease shows a reversed or null gradient [9], suggesting that associations with workplace size may differ across subtypes. Treating CVD as a monolithic category could mask or distort these patterns. Evidence on workplace-specific CVD subtype associations remains sparse [10], and existing studies have relied on risk factor measures rather than diagnosed disease, used limited samples, and employed narrow size classifications (typically two to four categories). No investigation has used large-scale administrative claims data to examine multiple CVD subtypes simultaneously across workplace size categories aligned with regulatory thresholds. Without subtype-specific evidence, workplace health policies risk applying uniform interventions to heterogeneous disease burdens.

We therefore examined the association between workplace size and the prevalence of 10 CVD subtypes among 11.2 million Korean wage workers, using National Health Insurance Service administrative claims data, to determine whether recorded prevalence patterns were uniform or subtype-specific.

## Methods

### Study design and data sources

We conducted a nationwide cross-sectional study of wage workers in South Korea using the National Health Insurance Service (NHIS) database. The NHIS is a mandatory single-payer system covering virtually the entire population of approximately 52 million residents, providing near-complete capture of both workforce and healthcare utilization data [11]. Three databases were linked using encrypted personal identifiers assigned by the NHIS: the eligibility database (demographic and insurance qualification records), the claims database (ICD-10-coded principal diagnoses and medical service utilization during 2021), and the National Tax Service income database (annual earnings, 2020) [12]. The analysis therefore estimated recorded CVD prevalence during 2021 rather than disease incidence.

### Study population

The study population comprised individuals who, as of 2021, were (1) enrolled in workplace-based health insurance, (2) aged 15–64 years, (3) Korean nationals, (4) recipients of wage income only, with no recorded business or miscellaneous income, and (5) earning an annual wage income of at least 10,000 KRW (approximately 7 USD) to exclude nominal records.

We required linkage to firm-level data and a valid nonzero workplace-size record. Among 11,345,158 wage-income-only workers, 131,782 were not linked to firm-level data. Among linked workers, no records were excluded for missing or zero workplace size, and 864 were excluded because industry code was missing. For those employed at multiple workplaces, the primary workplace was determined hierarchically: first by the longest employment duration, then by the largest workforce, and finally by the lowest workplace identifier. The final analytic sample consisted of 11,212,512 wage workers (Additional file 1: Fig. S1).

### Exposure

The exposure variable was the number of regular workers at the primary workplace, categorized into eight groups aligned with regulatory thresholds under the Korean Occupational Safety and Health Act: 1, 2–4, 5–29, 30–49, 50–99, 100–299, 300–999, and 1000 or more workers. These thresholds mark successive tiers of mandatory occupational health and safety obligations (at 5, 30, 50, 100, 300, and 1000 workers). Workplaces with fewer than five workers are additionally exempt from most provisions of the Labor Standards Act, including regulations on working hours, paid leave, and unfair dismissal. The 5–29-worker category was chosen as the reference because it represents the first tier at which full labor standards apply and constitutes the largest single stratum (30.5% of the study population).

### Outcome

CVD cases were identified from the claims database using the principal diagnosis field. Claims before 2021 were not used to identify pre-existing CVD, so the outcomes represent CVD recorded during the 2021 observation period rather than incident first-ever disease. For each of the 10 subtypes defined by ICD-10 chapter blocks (Table 2), an individual was classified as a case if three or more claim records (outpatient or inpatient) with the corresponding diagnosis code were recorded during 2021; claims from dental and traditional Korean medicine services were excluded. This threshold is more conservative than commonly used Korean claims-based cardiovascular definitions, such as one hospitalization or two outpatient visits [12, 13]. In NHIS atrial fibrillation research, a less restrictive definition using discharge diagnosis or at least two outpatient diagnoses showed a positive predictive value of 94.1% [14]. We used the three-claim threshold to improve specificity for subtype-specific analysis, recognizing that this stricter definition may reduce sensitivity, particularly for participants with only one or two relevant records or disease first recorded late in 2021. The 10 subtypes encompassed hypertensive diseases (I10–I15), ischemic heart diseases (IHD; I20–I25), cerebrovascular diseases (I60–I69), and seven additional groups spanning I00–I99 (Table 2; Additional file 1: Table S1).

### Statistical analysis

Baseline characteristics were described by workplace size category using frequencies with percentages and means with standard deviations (SD).

Crude prevalence per 100,000 workers was calculated with Wald 95% confidence intervals (CIs). Directly standardized prevalence was computed using the sex-by-age distribution of the entire study population (20 strata) as the internal standard, with variance estimated by the Keyfitz formula [15].

Logistic regression models estimated odds ratios (ORs) with 95% CIs for CVD prevalence across workplace size categories. ORs were chosen as the effect measure for comparability with the existing literature on workplace size and CVD; for hypertensive diseases (prevalence approximately 9%), ORs may overestimate prevalence ratios owing to non-collapsibility, a limitation addressed in the Discussion. Both unadjusted and adjusted ORs (aORs) were computed for each CVD subcategory; the adjusted model included sex and age (10 five-year groups from 15 to 19 to 60–64 years). The adjusted model was limited by the available administrative variables and did not include behavioral or clinical risk factors such as smoking, alcohol consumption, body mass index, physical activity, working hours, or psychosocial stress. Three sensitivity analyses redefined CVD cases using hospitalization-based criteria, requiring a cumulative total of at least 1, 3, or 5 inpatient days within each subcategory during 2021. Because these more restrictive definitions substantially reduced case counts — particularly in smaller workplace-size categories — the sex-by-age-by-workplace-size cells required for logistic regression became too sparse to yield stable adjusted estimates; crude odds ratios are therefore reported for the sensitivity analyses.

As a supplementary analysis, the adjusted model was additionally controlled for industry type (21 categories based on the Korean Standard Industrial Classification) to evaluate potential confounding by industrial composition. E-values were calculated for all statistically significant associations to quantify the minimum confounder strength required to explain away each observed association [16].

Given the exploratory nature of subtype-specific analyses across multiple workplace size categories, no correction for multiple comparisons was applied. Accordingly, findings for subtypes with low case counts should be interpreted as hypothesis-generating rather than confirmatory.

All statistical analyses were performed with SAS 9.4 (SAS Institute, Cary, NC, USA), and figures were produced with R 4.5.2 (R Foundation for Statistical Computing, Vienna, Austria). Two-sided P values below 0.05 were considered statistically significant.

## Results

### Study population

The study comprised 11,212,512 workers (Additional file 1: Fig. S1): 6,260,083 men (55.8%) and 4,952,429 women (44.2%), with a mean age of 41.7 years (SD 11.9). The 5–29-person category formed the largest stratum (*n* = 3,422,154; 30.5%), whereas one-person workplaces represented the smallest (*n* = 89,227; 0.8%) (Table 1).

**Table 1:** Baseline characteristics of study participants by workplace size

Variable	1	2–4	5–29	30–49	50–99	100–299	300–999	≥ 1,000	Total
*N*	89,227	1,183,181	3,422,154	829,052	959,997	1,370,392	1,105,375	2,253,134	11,212,512
Sex, *n* (%)									
Male	52,828 (59.2)	581,475 (49.1)	1,723,964 (50.4)	452,159 (54.5)	539,924 (56.2)	825,421 (60.2)	684,961 (62.0)	1,399,351 (62.1)	6,260,083 (55.8)
Female	36,399 (40.8)	601,706 (50.9)	1,698,190 (49.6)	376,893 (45.5)	420,073 (43.8)	544,971 (39.8)	420,414 (38.0)	853,783 (37.9)	4,952,429 (44.2)
Age, mean (SD)	46.1 (11.3)	43.2 (11.9)	42.8 (12.0)	43.1 (12.4)	42.6 (12.3)	41.4 (11.9)	40.0 (11.7)	39.2 (11.1)	41.7 (11.9)
15–24	4,004 (4.5)	76,946 (6.5)	226,403 (6.6)	53,284 (6.4)	61,864 (6.4)	90,674 (6.6)	79,302 (7.2)	177,615 (7.9)	770,092 (6.9)
25–34	11,700 (13.1)	234,896 (19.9)	748,567 (21.9)	189,151 (22.8)	233,933 (24.4)	372,352 (27.2)	344,425 (31.2)	699,502 (31.0)	2,834,526 (25.3)
35–44	20,016 (22.4)	291,466 (24.6)	823,435 (24.1)	189,423 (22.8)	223,948 (23.3)	343,135 (25.0)	287,672 (26.0)	616,705 (27.4)	2,795,800 (24.9)
45–54	29,902 (33.5)	335,737 (28.4)	924,922 (27.0)	206,274 (24.9)	232,826 (24.3)	321,509 (23.5)	231,994 (21.0)	517,698 (23.0)	2,800,862 (25.0)
55–64	23,605 (26.5)	244,136 (20.6)	698,827 (20.4)	190,920 (23.0)	207,426 (21.6)	242,722 (17.7)	161,982 (14.7)	241,614 (10.7)	2,011,232 (17.9)
Income, mean (SD)ᵃ	2,796 (2,560)	2,473 (2,069)	3,299 (2,889)	3,770 (3,399)	3,963 (3,604)	4,468 (3,838)	5,204 (4,273)	6,697 (5,006)	4,313 (3,949)
Median income income	2,220	2,200	2,753	3,161	3,288	3,747	4,404	5,760	3,655

ᵃ Income in 10,000 Korean Won (KRW)

*Abbreviation: SD *standard deviation

Worker characteristics varied substantially across workplace size categories (Table 1). Workers in one-person workplaces (mean age 46.1 years) were nearly seven years older on average than those in the largest workplaces (39.2 years). The proportion of men was lowest in 2–4-person workplaces (49.1%) and highest in the 1000-or-more category (62.1%), with one-person workplaces (59.2%) deviating from the otherwise ascending gradient. Median annual income differed more than 2.5-fold between one-person workplaces (22.2 million KRW) and those with 1000 or more workers (57.6 million KRW).

### Prevalence of cardiovascular diseases

**Fig. 1:**
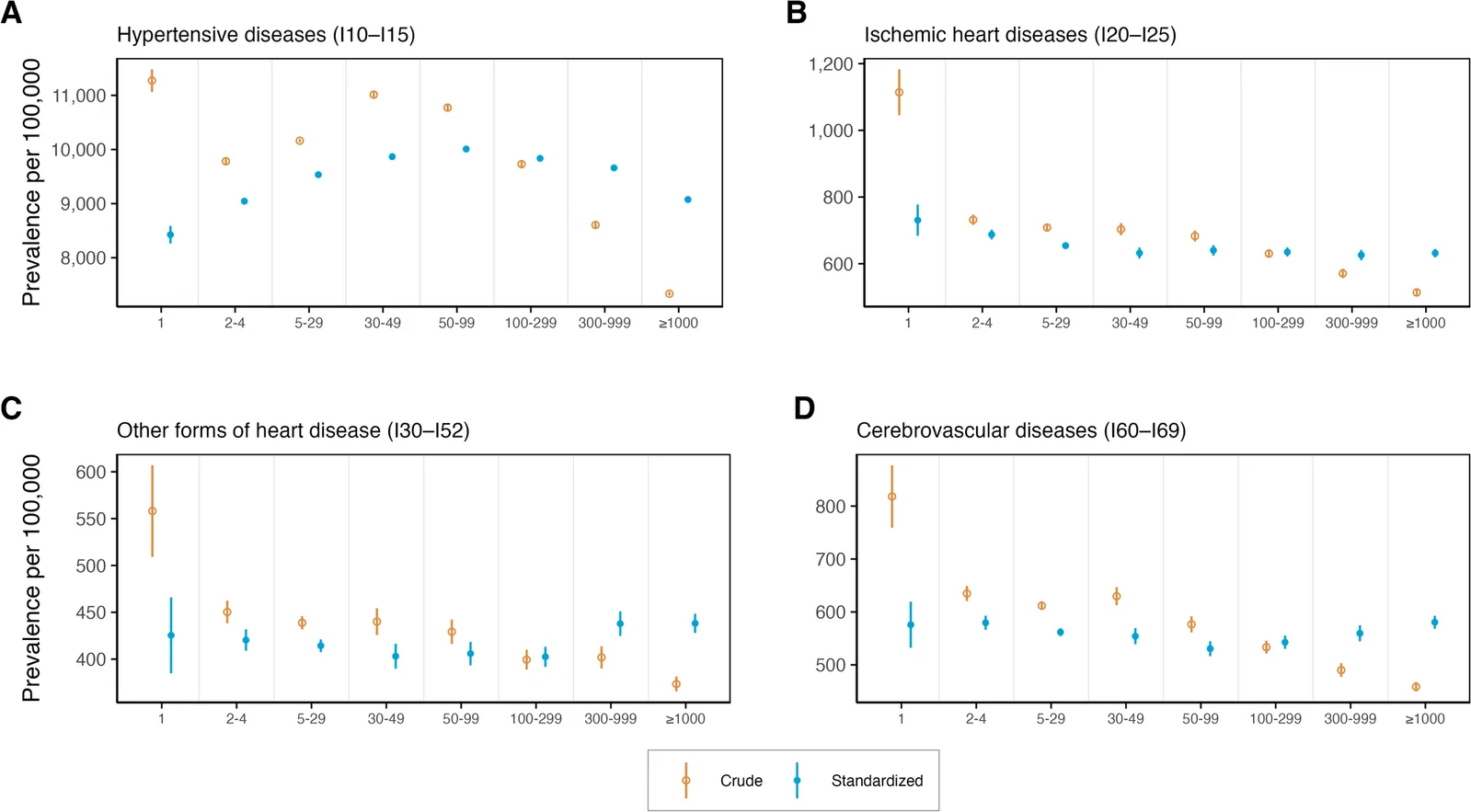
Crude and age- and sex-standardized prevalence of major cardiovascular diseases by workplace size. **A** Hypertensive diseases (I10–I15); **B** Ischemic heart diseases (I20–I25); **C** Other forms of heart disease (I30–I52); **D** Cerebrovascular diseases (I60–I69). Open circles indicate crude prevalence; filled circles indicate standardized prevalence. Error bars represent 95% confidence intervals. Rates per 100,000 workers

Among the four major CVD categories, hypertensive diseases (I10–I15) had the highest overall crude prevalence (9,471.5 per 100,000 workers), followed by IHD (I20–I25; 649.4), cerebrovascular diseases (I60–I69; 561.8), and other forms of heart disease (I30–I52; 418.6) (Table 2, Fig. 1).

**Table 2:** Prevalence of major cardiovascular diseases by workplace size (per 100,000)

Disease	1	2–4	5–29	30–49	50–99	100–299	300–999	≥ 1,000	Totalᵃ
Hypertensive diseases (I10–I15)									
Crude rate (95% CI)	11,273.5 (11,053.2–11,493.8)	9,782.5 (9,726.2–9,838.9)	10,161.8 (10,128.0–10,195.6)	11,013.7 (10,942.2–11,085.1)	10,773.0 (10,707.3–10,838.6)	9,728.8 (9,676.6–9,781.0)	8,605.6 (8,550.9–8,660.3)	7,334.7 (7,299.4–7,370.1)	9,471.5 (9,453.5–9,489.5)
Std. rate (95% CI)	8,425.8 (8,264.3–8,587.3)	9,044.6 (8,996.3–9,093.0)	9,534.6 (9,505.5–9,563.7)	9,867.0 (9,807.8–9,926.1)	10,008.3 (9,952.4–10,064.3)	9,835.4 (9,787.5–9,883.2)	9,660.6 (9,604.3–9,716.9)	9,075.6 (9,032.7–9,118.5)	9,471.5 (9,453.5–9,489.5)ᵇ
IHD (I20–I25)									
Crude rate (95% CI)	1,114.0 (1,044.8–1,183.3)	731.8 (716.4–747.3)	708.5 (699.6–717.4)	703.5 (685.4–721.5)	683.0 (666.5–699.6)	630.5 (617.2–643.8)	571.0 (556.9–585.1)	513.9 (504.5–523.3)	649.4 (644.7–654.2)
Std. rate (95% CI)	730.5 (683.8–777.3)	687.6 (673.1–702.0)	654.2 (646.0–662.5)	632.2 (615.9–648.4)	640.2 (624.7–655.7)	635.3 (622.0–648.6)	625.9 (610.5–641.4)	631.8 (619.2–644.4)	649.4 (644.7–654.2)ᵇ
Other forms of heart disease (I30–I52)									
Crude rate (95% CI)	558.1 (509.1–607.1)	450.3 (438.2–462.4)	438.8 (431.8–445.8)	440.0 (425.7–454.3)	429.2 (416.1–442.3)	399.4 (388.9–410.0)	401.9 (390.0–413.7)	373.5 (365.5–381.5)	418.6 (414.9–422.4)
Std. rate (95% CI)	425.5 (385.0–466.0)	420.4 (408.9–431.8)	414.3 (407.6–421.0)	403.1 (389.9–416.4)	406.0 (393.4–418.5)	402.5 (391.8–413.1)	437.9 (424.8–450.9)	438.2 (427.9–448.5)	418.6 (414.9–422.4)ᵇ
Cerebrovascular diseases (I60–I69)									
Crude rate (95% CI)	818.1 (758.8–877.5)	634.8 (620.5–649.2)	611.8 (603.5–620.1)	629.6 (612.6–646.7)	576.6 (561.4–591.8)	533.5 (521.3–545.7)	490.2 (477.1–503.2)	458.7 (449.8–467.5)	561.8 (557.5–566.2)
Std. rate (95% CI)	575.8 (532.3–619.3)	579.6 (566.4–592.8)	561.8 (554.2–569.5)	554.3 (539.0–569.5)	530.6 (516.5–544.7)	542.8 (530.4–555.2)	559.7 (544.6–574.7)	580.6 (568.3–592.8)	561.8 (557.5–566.2)ᵇ

Rates per 100,000 workers

Crude rate: Wald 95% CI. Std. rate: age- and sex-standardized to the total study population

ᵃ For the Total column, the standardized rate is replaced by the crude rate. The population-weighted mean of size-specific standardized rates does not constitute a standard epidemiological measure; direct standardization of the entire study population to its own age–sex distribution yields the crude rate

*Abbreviations: CI *confidence interval, *Std. *standardized

The contrast between crude and standardized rates was most striking for hypertensive diseases (Table 2, Fig. 1). Crude rates were highest in one-person workplaces (11,273.5 per 100,000) and lowest in the 1000-or-more category (7,334.7) — a 54% relative difference. After standardization, however, this gradient was substantially attenuated: rates rose from 8,425.8 in one-person workplaces to 10,008.3 in the 50–99 category before declining to 9,075.6 in the 1000-or-more category, reshaping a monotonic decline into an inverted-U pattern. IHD standardized rates remained highest in one-person workplaces (730.5), whereas cerebrovascular standardized rates were highest in the 1000-or-more category (580.6) without a monotonic trend. Standardized rates for other forms of heart disease ranged narrowly from 402.5 (100–299) to 438.2 (1000 or more).

The remaining six CVD categories had considerably lower prevalence. Diseases of veins and lymphatic vessels (I80–I89) were most common (standardized: 304.9 per 100,000), followed by diseases of arteries (I70–I79; 158.6). The four remaining categories — acute rheumatic fever, chronic rheumatic heart diseases, pulmonary heart disease, and other circulatory disorders — each had standardized rates at or below 15.1 per 100,000 (Additional file 1: Table S1 and Fig. S2).

### Association between workplace size and cardiovascular disease subtypes.

**Fig. 2:**
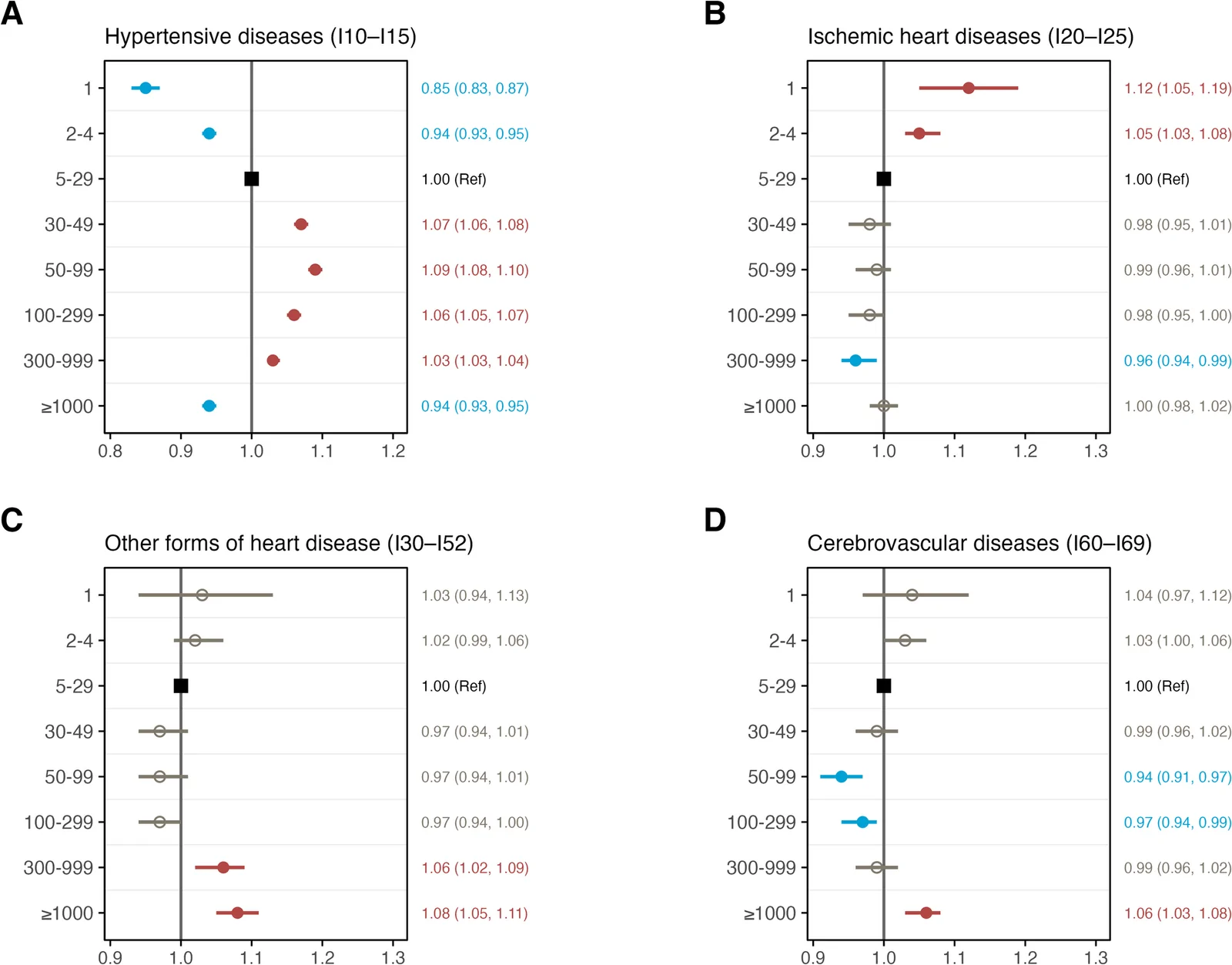
Adjusted odds ratios with 95% confidence intervals for major cardiovascular diseases by workplace size. **A** Hypertensive diseases (I10–I15); **B** Ischemic heart diseases (I20–I25); **C** Other forms of heart disease (I30–I52); **D** Cerebrovascular diseases (I60–I69). Reference category: workplace size 5–29 workers. Adjusted for sex and age (5-year groups)

Table 3 and Fig. 2 present age- and sex-adjusted odds ratios with the 5–29-person category as reference. The four major CVD subtypes showed different patterns across workplace-size categories.

**Table 3:** Odds ratios for major cardiovascular diseases by workplace size

Disease / Workplace size	Crude OR (95% CI)	Adjusted OR (95% CI)
Hypertensive diseases (I10–I15)		
1	1.12 (1.10–1.15)	0.85 (0.83–0.87)*
2–4	0.96 (0.95–0.97)	0.94 (0.93–0.95)*
5–29 (Ref)	1.00	1.00
30–49	1.09 (1.09–1.10)	1.07 (1.06–1.08)*
50–99	1.07 (1.06–1.08)	1.09 (1.08–1.10)*
100–299	0.95 (0.95–0.96)	1.06 (1.05–1.07)*
300–999	0.83 (0.83–0.84)	1.03 (1.03–1.04)*
≥ 1,000	0.70 (0.70–0.70)	0.94 (0.93–0.95)*
IHD (I20–I25)		
1	1.58 (1.48–1.68)	1.12 (1.05–1.19)*
2–4	1.03 (1.01–1.06)	1.05 (1.03–1.08)*
5–29 (Ref)	1.00	1.00
30–49	0.99 (0.96–1.02)	0.98 (0.95–1.01)
50–99	0.96 (0.94–0.99)	0.99 (0.96–1.01)
100–299	0.89 (0.87–0.91)	0.98 (0.95–1.00)
300–999	0.80 (0.78–0.83)	0.96 (0.94–0.99)*
≥ 1,000	0.72 (0.71–0.74)	1.00 (0.98–1.02)
Other forms of heart disease (I30–I52)		
1	1.27 (1.16–1.39)	1.03 (0.94–1.13)
2–4	1.03 (0.99–1.06)	1.02 (0.99–1.06)
5–29 (Ref)	1.00	1.00
30–49	1.00 (0.97–1.04)	0.97 (0.94–1.01)
50–99	0.98 (0.94–1.01)	0.97 (0.94–1.01)
100–299	0.91 (0.88–0.94)	0.97 (0.94–1.00)
300–999	0.92 (0.89–0.95)	1.06 (1.02–1.09)*
≥ 1,000	0.85 (0.83–0.87)	1.08 (1.05–1.11)*
Cerebrovascular diseases (I60–I69)		
1	1.34 (1.24–1.44)	1.04 (0.97–1.12)
2–4	1.04 (1.01–1.07)	1.03 (1.00–1.06)
5–29 (Ref)	1.00	1.00
30–49	1.03 (1.00–1.06)	0.99 (0.96–1.02)
50–99	0.94 (0.91–0.97)	0.94 (0.91–0.97)*
100–299	0.87 (0.85–0.89)	0.97 (0.94–0.99)*
300–999	0.80 (0.78–0.82)	0.99 (0.96–1.02)
≥ 1,000	0.75 (0.73–0.77)	1.06 (1.03–1.08)*

Crude OR: unadjusted odds ratio. Adjusted OR: adjusted for sex and age (5-year groups). Reference category: workplace size 5–29 workers

*Statistically significant (95% CI excludes 1.00)

*Abbreviations: OR *odds ratio, *CI *confidence interval

For hypertensive diseases, the crude OR for one-person workplaces was 1.12 (95% CI 1.10–1.15), but after adjustment the aOR was 0.85 (0.83–0.87). All four mid-range categories (30–49 through 300–999 persons) had aORs above 1.00, peaking at 1.09 (1.08–1.10) in the 50–99-person category, while both extremes (one-person and 1000-or-more) showed aORs below 1.00.

IHD showed a contrasting pattern. aORs were elevated in the smallest workplaces — 1.12 (1.05–1.19) for one-person and 1.05 (1.03–1.08) for 2–4-person — and lower in the 300–999-person category (0.96; 0.94–0.99) (Table 3, Fig. 2). The remaining mid-range and large categories showed aORs ranging from 0.98 to 1.00, all with 95% CIs including 1.00.

Cerebrovascular diseases and other forms of heart disease (I30–I52) diverged from both hypertension and IHD, with elevated aORs concentrated in large workplaces. Other forms of heart disease showed elevated aORs only in the two largest categories (300–999: 1.06, 1.02–1.09; 1000 or more: 1.08, 1.05–1.11). Cerebrovascular aORs were below 1.00 in 50–99-person (0.94; 0.91–0.97) and 100–299-person workplaces (0.97; 0.94–0.99) and above 1.00 in the 1000-or-more category (1.06; 1.03–1.08).

Results for the six remaining CVD categories are detailed in Additional file 1: Table S2 and Fig. S3.

### Sensitivity analyses

Hospitalization-based case definitions (at least 1, 3, and 5 inpatient days) yielded lower prevalence rates than the claims-based definition (Additional file 1: Tables S3–S5).

For IHD, crude ORs for one-person workplaces remained elevated under each hospitalization-based definition (at least 1 day: 1.50, 95% CI 1.32–1.70; at least 3 days: 1.50, 1.31–1.71; at least 5 days: 1.57, 1.37–1.80) (Additional file 1: Tables S6–S8).

Cerebrovascular diseases showed different patterns across case definitions. Under the claims-based definition, adjusted odds were elevated in the largest workplaces. Under the 5-or-more-day hospitalization criterion, directly standardized rates were highest in one-person workplaces (163.6 per 100,000) and lowest in the 1000-or-more category (115.1). Cerebrovascular crude ORs for one-person workplaces were above 1.00 under each hospitalization criterion, whereas those for 1000-or-more workplaces were below 1.00, all with 95% CIs excluding 1.00.

In the supplementary analysis additionally adjusting for 21 industry categories (Additional file 1: Table S9), aORs for hypertensive diseases, IHD, and other forms of heart disease were virtually unchanged (maximum absolute change: 0.02). For cerebrovascular diseases, the aOR for the largest workplaces was attenuated from 1.06 to 1.01 and lost statistical significance after industry adjustment.

E-values for all statistically significant associations are reported in Additional file 1: Table S10. For the two associations of greatest interest — hypertensive diseases in one-person workplaces (aOR 0.85, E-value 1.58) and IHD in one-person workplaces (aOR 1.12, E-value 1.48) — an unmeasured confounder would need to be associated with both workplace size and the outcome by a risk ratio of at least this magnitude to fully explain the observed associations.

## Discussion

In this nationwide cross-sectional analysis of 11.2 million Korean wage workers, recorded CVD prevalence differed by subtype across workplace-size categories. Hypertensive diseases showed a crude excess in one-person workplaces that changed direction after adjustment for age and sex, while IHD showed modestly elevated adjusted odds in the smallest workplaces and cerebrovascular diseases showed modestly elevated adjusted odds in the largest workplaces. This analysis is designed as descriptive cardiovascular surveillance rather than an etiological investigation: it maps the distribution of recorded CVD prevalence across administrative workplace-size categories but does not test causal hypotheses or establish temporal ordering. The findings should therefore be interpreted as prevalence patterns rather than evidence of causal effects.

The hypertension findings illustrate the importance of separating crude differences from adjusted prevalence patterns. Workers in the smallest workplaces were older on average, which likely contributed to their high crude hypertension prevalence. After age and sex adjustment, the highest hypertension aORs appeared in mid-sized workplaces, whereas one-person and very large workplaces had aORs below 1.00. One possible explanation is differential detection: hypertension is often asymptomatic and may be more completely identified where workplace health-screening access and follow-up are more available. Prior Korean evidence suggests that health-screening participation differs by workplace size [17]. However, screening participation was not measured directly in the present analysis, so screening-related ascertainment should be regarded as a plausible explanation rather than a demonstrated mechanism.

The inverted-U pattern of hypertensive disease aORs is consistent with differential screening access across workplace sizes. Although all insured Korean workers are eligible for biennial health screening, participation rates vary by workplace size, with lower uptake in the smallest workplaces [17]. The Occupational Safety and Health Act additionally mandates that workplaces with 50 or more employees appoint occupational health professionals, which may facilitate more systematic screening and follow-up in mid-range workplaces and contribute to the higher adjusted prevalence observed in that range.

The IHD pattern differed from the hypertension pattern. The aORs for one-person and 2–4-person workplaces were elevated but small in magnitude (1.12 and 1.05, respectively), and the large sample size means that statistical significance should not be equated with large individual-level risk differences. At the population level, however, even modest differences may be relevant when they occur in worker groups with limited statutory protections. Hospitalization-based sensitivity analyses showed elevated crude ORs for IHD in one-person workplaces under increasingly restrictive definitions, but these analyses remain descriptive and cannot address whether the pattern reflects greater disease severity, differential ascertainment, or health-related worker selection.

The contrast with IHD provides interpretive context. IHD manifests symptomatically and is therefore less dependent on proactive screening for diagnosis. Its pattern of elevated adjusted prevalence in the smallest workplaces — opposite to hypertension — is less likely to reflect differential ascertainment and more likely to reflect a genuine difference in recorded disease burden, though residual confounding cannot be excluded.

The cerebrovascular findings also require caution. In the main claims-based analysis, the largest workplaces showed a modestly elevated aOR, whereas hospitalization-based standardized rates were highest in one-person workplaces under the most restrictive definition. This divergence may reflect differences between recorded outpatient/inpatient claims and more severe hospitalized events, but it may also reflect residual confounding, case-mix differences, or health-related worker movement. One possible contributor to the elevated cerebrovascular aOR in the largest workplaces is differential worker retention: large employers may retain workers with cerebrovascular disease through structured disability management or greater job security, whereas affected workers at smaller workplaces may be more likely to exit the workforce. The present cross-sectional design cannot distinguish these possibilities.

The heterogeneity of associations across subtypes has a biological basis. CVD subtypes differ in their risk factor profiles; in East Asian populations, hypertension is more strongly associated with cerebrovascular disease than IHD, whereas hyperlipidaemia and diabetes show stronger links to IHD [18]. Large-scale data further show that the relationship between blood pressure and incidence of CVD varies substantially across 12 subtypes [19]. In the Korean population, stroke and IHD show divergent socioeconomic gradients [9], a pattern consistent with our finding of opposite workplace-size associations for these two subtypes. Non-uniform CVD associations are therefore expected, given that age structure, income, and working conditions differ across workplace-size categories [8].

Workplace size should therefore be treated as an administrative and organizational marker, not as a measured mechanism. Smaller enterprises may differ from larger ones in occupational hazards and organizational protection resources [20, 21], health-promotion capacity [22, 23], working hours, and psychosocial working conditions [24]. Recent national evidence from Korean manufacturing workers showed substantial enterprise-size disparities in workplace hazards and organizational protection resources [25]. Among potential structural pathways, long working hours are particularly relevant: Korean enterprises with fewer than five workers are exempt from statutory working-hour limits [5], and long working hours have been associated with elevated risks of both stroke and coronary heart disease in a pooled analysis of 603,838 individuals [26]. Because the present study did not include direct measures of these pathways, such factors remain hypotheses that may help interpret the observed patterns rather than variables tested in the analysis.

The hospitalization-based sensitivity analyses provide additional descriptive context. The persistence and modest strengthening of IHD crude ORs in one-person workplaces under increasingly stringent definitions (≥ 1 day: 1.50, 95% CI 1.32–1.70; ≥3 days: 1.50, 1.31–1.71; ≥5 days: 1.57, 1.37–1.80) is consistent with a pattern less attributable to incidental diagnoses, though adjusted estimates could not be computed for these definitions, as the more restrictive hospitalization criteria substantially reduced case counts — particularly in smaller workplace-size categories — leaving several sex-by-age-by-workplace-size strata too sparse for stable logistic regression estimation. For cerebrovascular diseases, the divergence between claims-based and hospitalization-based patterns may reflect differences in case mix between mild detected events and severe hospitalized events, but this interpretation remains speculative in the absence of adjusted hospitalization estimates.

A supplementary analysis adjusting for 21 major industry categories confirmed that aORs were virtually unchanged for hypertensive diseases, IHD, and other forms of heart disease (maximum absolute change: 0.02). For cerebrovascular diseases, however, the aOR for the largest workplaces was attenuated from 1.06 to 1.01 and lost statistical significance after industry adjustment, suggesting partial confounding by industrial composition for this specific subtype–size combination (Additional file 1: Table S9).

E-values provide a formal framework for quantifying vulnerability to residual confounding [16]; full results are reported in Additional file 1: Table S10. The E-value for a given association is the minimum strength of association — expressed on the risk ratio scale — that an unmeasured confounder would need to have with both the exposure and the outcome simultaneously to fully explain away the observed association; lower E-values indicate greater vulnerability to unmeasured confounding. The thresholds for the two associations of greatest interest are modest: 1.58 for hypertensive diseases in one-person workplaces (aOR 0.85) and 1.48 for IHD in one-person workplaces (aOR 1.12). Common cardiovascular risk factors such as smoking (relative risk for IHD approximately 2–3) exceed these thresholds, indicating that an unmeasured confounder of plausible magnitude could explain the observed associations. These results reinforce the interpretation of the findings as descriptive prevalence patterns rather than causal estimates.

These findings have practical implications for workplace-based cardiovascular surveillance. Workplace size is routinely recorded in national health insurance databases and could serve as a stratification variable to monitor differential CVD prevalence across worker populations. The concentration of diagnosed hypertension in mid-range workplaces and the elevated IHD prevalence in the smallest workplaces represent subgroup patterns that may be relevant to the design and targeting of workplace-based cardiovascular surveillance programs. In particular, the smallest workplaces — where statutory protections for working hours and occupational health services are limited — may represent a population that is currently underrepresented in routine cardiovascular surveillance — a gap that existing Korean occupational disease surveillance infrastructure has demonstrated capacity to address through enhanced case detection [27].

The main analytical limitation is residual confounding. The NHIS administrative data used here did not include smoking, alcohol consumption, obesity, physical activity, working hours, occupational exposures, psychosocial stress, or clinical risk-factor measurements. These factors are plausibly associated with both workplace size and CVD, and their unavailability is a shared constraint in Korean occupational studies that rely on administrative claims data [28, 29, 30]. Therefore, the age- and sex-adjusted ORs should not be interpreted as fully confounder-adjusted estimates. In an etiological study, this would constitute a fundamental limitation precluding causal inference. In the present surveillance context, however, the observed prevalence patterns across workplace-size categories retain independent descriptive value as indicators of where cardiovascular disease burden is concentrated in the working population — information that is relevant for surveillance design and resource prioritization regardless of the underlying causal mechanisms.

Several additional limitations follow from the study design and outcome definition. First, the analysis used cross-sectional prevalence data, so temporal relationships cannot be established. We could not determine how long workers had been employed in the same company, industry, or workplace-size category before CVD was recorded. Health-related job changes, including movement of workers with existing CVD into smaller workplaces or self-employment, may have contributed to the observed patterns. Second, the outcome was based on claims recorded in 2021 only. Pre-existing CVD before 2021 was not explicitly identified unless the participant also had at least three qualifying claims during 2021. Third, the requirement of three or more claims improves specificity but may reduce sensitivity, particularly for cases first recorded late in 2021 or for participants with only one or two relevant diagnostic records. Fourth, the study did not directly measure health-screening participation by workplace size, limiting inference about detection bias. Finally, ORs may overstate prevalence ratios for common outcomes such as hypertension, and many statistically significant estimates were close to the null.

This study also has strengths. The sample includes nearly the entire Korean wage-worker population in 2021, avoiding selection bias from voluntary survey participation and providing sufficient power to examine multiple CVD subtypes. The use of administrative claims avoids self-reporting of disease status. The simultaneous evaluation of 10 CVD subtypes across regulatory workplace-size categories shows that aggregate CVD measures may conceal heterogeneous prevalence patterns.

Taken together, these results suggest that workplace-size categories can be useful for descriptive surveillance of cardiovascular health inequalities, but they should not be interpreted as evidence that workplace size itself causes subtype-specific CVD risks. Future longitudinal studies linking employment histories, health-screening participation, behavioral risk factors, clinical measurements, and detailed occupational exposures are needed to determine whether the observed prevalence patterns reflect causal pathways, detection differences, worker selection, or a combination of these processes.

## Conclusions

The association between workplace size and CVD prevalence was heterogeneous across subtypes. Hypertensive disease showed the highest adjusted prevalence in mid-range workplaces, a pattern consistent with demographic composition differences, whereas IHD and cerebrovascular diseases showed divergent prevalence patterns across workplace sizes. These findings indicate that the relationship between workplace size and CVD prevalence varies by subtype, with no uniform pattern across all workplace-size categories, and support disease-specific approaches to workplace-based cardiovascular surveillance. Because adjustment was limited to age and sex, these associations should be interpreted as descriptive prevalence patterns rather than causal effects. 

## Supplementary Information

Supplementary Material 1.

## Data Availability

The datasets used during the current study were obtained from the National Health Insurance Service under a data use agreement (NHIS-2025-07-1-069). Restrictions apply to the availability of these data, which contain sensitive personal health information. Data access requests can be submitted to the National Health Insurance Service (https://nhiss.nhis.or.kr).

## Abbreviations

aOR: Adjusted odds ratio
CI: Confidence interval
CVD: Cardiovascular disease
ICD-10: International Classification of Diseases, 10th Revision
IHD: Ischemic heart disease
NHIS: National Health Insurance Service
OR: Odds ratio
SD: Standard deviation
