# Supplementary material for: Workplace size and cardiovascular disease subtypes among 11 million Korean wage workers: a nationwide cross-sectional study
Source: BMC Public Health. 2026 Jul 8;26:2726. doi: 10.1186/s12889-026-28385-y (PMC13628708; doi:10.1186/s12889-026-28385-y)
Supplement: Supplementary file 1 [file 12889_2026_28385_MOESM1_ESM.docx]

**Additional file 1**

Workplace size and cardiovascular disease subtypes among 11 million Korean wage workers: a nationwide cross-sectional study

Yangwoo Kim, Minji Koo, Eun Mi Kim, Jaiyong Kim, Inah Kim

**Contents**

Fig. S1 Flow chart of study population selection from the National Health Insurance Service database, 2021

Table S1 Prevalence of other cardiovascular diseases by workplace size (per 100000)

Table S2 Odds ratios for other cardiovascular diseases by workplace size

Fig. S2 Crude and age- and sex-standardized prevalence of other cardiovascular diseases by workplace size

Fig. S3 Adjusted odds ratios with 95% confidence intervals for other cardiovascular diseases by workplace size

Table S3 Sensitivity analysis: prevalence of cardiovascular diseases by workplace size, IPD ≥1 day (per 100000)

Table S4 Sensitivity analysis: prevalence of cardiovascular diseases by workplace size, IPD ≥3 days (per 100000)

Table S5 Sensitivity analysis: prevalence of cardiovascular diseases by workplace size, IPD ≥5 days (per 100000)

Table S6 Sensitivity analysis: crude odds ratios for cardiovascular diseases by workplace size, IPD ≥1 day

Table S7 Sensitivity analysis: crude odds ratios for cardiovascular diseases by workplace size, IPD ≥3 days

Table S8 Sensitivity analysis: crude odds ratios for cardiovascular diseases by workplace size, IPD ≥5 days

Table S9 Adjusted odds ratios for cardiovascular disease subtypes by workplace size, adjusted for industry type

Table S10 E-values for the association between workplace size and cardiovascular disease subtypes

**Fig. S1** Flow chart of study population selection from the National Health Insurance Service database, 2021

**Table S1** Prevalence of other cardiovascular diseases by workplace size (per 100000)

| **Disease** | **1** | **2–4** | **5–29** | **30–49** | **50–99** | **100–299** | **300–999** | **≥1000** | **Total** |
| --- | --- | --- | --- | --- | --- | --- | --- | --- | --- |
| **Acute rheumatic fever (I00–I02)** | | | | | | | | | |
| Crude rate (95% CI) | 2.2 (0.0–5.3) | 0.6 (0.2–1.0) | 0.5 (0.2–0.7) | 0.7 (0.1–1.3) | 0.6 (0.1–1.1) | 0.4 (0.0–0.7) | 0.5 (0.1–0.8) | 0.4 (0.1–0.7) | 0.5 (0.4–0.6) |
| Std. rate (95% CI) | 1.7 (-0.7–4.0) | 0.5 (0.1–0.9) | 0.4 (0.2–0.6) | 0.6 (0.1–1.1) | 0.6 (0.1–1.1) | 0.4 (0.0–0.7) | 0.5 (0.1–1.0) | 0.5 (0.1–0.9) | 0.5 (0.4–0.6) |
| **Chronic rheumatic heart diseases (I05–I09)** | | | | | | | | | |
| Crude rate (95% CI) | 23.5 (13.5–33.6) | 16.3 (14.0–18.6) | 17.3 (15.9–18.7) | 17.0 (14.2–19.8) | 14.0 (11.6–16.3) | 13.6 (11.7–15.6) | 11.5 (9.5–13.5) | 11.4 (10.0–12.8) | 14.7 (14.0–15.4) |
| Std. rate (95% CI) | 16.1 (9.0–23.2) | 14.1 (12.1–16.1) | 15.6 (14.4–16.9) | 14.6 (12.1–17.0) | 12.5 (10.4–14.7) | 14.2 (12.1–16.2) | 14.2 (11.7–16.7) | 15.4 (13.3–17.4) | 14.7 (14.0–15.5) |
| **Pulmonary heart disease (I26–I28)** | | | | | | | | | |
| Crude rate (95% CI) | 16.8 (8.3–25.3) | 12.3 (10.3–14.3) | 12.6 (11.4–13.8) | 12.8 (10.4–15.2) | 12.7 (10.5–15.0) | 12.3 (10.5–14.2) | 12.7 (10.6–14.8) | 11.5 (10.1–12.9) | 12.4 (11.7–13.0) |
| Std. rate (95% CI) | 14.0 (6.6–21.5) | 11.4 (9.5–13.3) | 12.1 (11.0–13.3) | 12.2 (9.8–14.6) | 12.4 (10.2–14.7) | 12.5 (10.6–14.4) | 13.6 (11.3–15.9) | 12.2 (10.6–13.8) | 12.3 (11.6–13.0) |
| **Diseases of arteries (I70–I79)** | | | | | | | | | |
| Crude rate (95% CI) | 224.1 (193.1–255.2) | 190.7 (182.8–198.5) | 177.3 (172.8–181.7) | 174.8 (165.8–183.8) | 160.6 (152.6–168.6) | 154.3 (147.7–160.8) | 135.5 (128.7–142.4) | 120.0 (115.5–124.5) | 159.0 (156.7–161.3) |
| Std. rate (95% CI) | 167.2 (142.9–191.5) | 171.2 (164.0–178.3) | 161.5 (157.4–165.6) | 150.6 (142.7–158.5) | 146.6 (139.2–154.0) | 158.0 (151.3–164.8) | 155.5 (147.5–163.5) | 157.1 (150.6–163.6) | 158.6 (156.2–161.0) |
| **Diseases of veins/lymphatic (I80–I89)** | | | | | | | | | |
| Crude rate (95% CI) | 272.3 (238.1–306.6) | 350.7 (340.0–361.3) | 320.3 (314.3–326.3) | 323.7 (311.5–336.0) | 316.7 (305.4–327.9) | 288.2 (279.2–297.2) | 277.7 (267.9–287.6) | 270.4 (263.6–277.2) | 304.9 (301.7–308.2) |
| Std. rate (95% CI) | 244.7 (211.4–277.9) | 313.9 (304.1–323.7) | 297.2 (291.5–302.8) | 300.0 (288.4–311.5) | 302.2 (291.4–313.1) | 301.6 (292.1–311.0) | 314.9 (303.4–326.3) | 314.4 (305.9–323.0) | 304.9 (301.6–308.3) |
| **Other circulatory disorders (I95–I99)** | | | | | | | | | |
| Crude rate (95% CI) | 24.7 (14.4–35.0) | 18.7 (16.2–21.1) | 15.8 (14.4–17.1) | 17.9 (15.0–20.7) | 15.3 (12.8–17.8) | 12.9 (11.0–14.8) | 14.5 (12.2–16.7) | 12.6 (11.1–14.0) | 15.1 (14.4–15.9) |
| Std. rate (95% CI) | 18.3 (10.3–26.4) | 16.9 (14.6–19.1) | 14.8 (13.5–16.0) | 16.2 (13.5–18.8) | 14.8 (12.4–17.2) | 13.2 (11.2–15.1) | 16.4 (13.8–19.1) | 14.8 (12.9–16.6) | 15.1 (14.4–15.8) |

Rates per 100000 workers. Crude rate: Wald 95% CI.

Std. rate: age- and sex-standardized to the total study population.

Abbreviations: CI, confidence interval; Std., standardized.

**Table S2** Odds ratios for other cardiovascular diseases by workplace size

| **Disease / Workplace size** | **Crude OR (95% CI)** | **Adjusted OR (95% CI)** |
| --- | --- | --- |
| **Acute rheumatic fever (I00–I02)** | | |
| 1 | 4.79 (1.10–20.85) | **4.68 (1.08–20.40)*** |
| 2–4 | 1.27 (0.52–3.08) | 1.21 (0.50–2.95) |
| 5–29 (Ref) | 1.00 | 1.00 |
| 30–49 | 1.55 (0.61–3.96) | 1.48 (0.58–3.79) |
| 50–99 | 1.34 (0.52–3.42) | 1.33 (0.52–3.41) |
| 100–299 | 0.78 (0.29–2.13) | 0.90 (0.33–2.45) |
| 300–999 | 0.97 (0.35–2.64) | 1.27 (0.46–3.49) |
| ≥1000 | 0.85 (0.38–1.93) | 1.22 (0.54–2.80) |
| **Chronic rheumatic heart diseases (I05–I09)** | | |
| 1 | 1.36 (0.88–2.11) | 1.17 (0.76–1.81) |
| 2–4 | 0.94 (0.80–1.11) | 0.91 (0.77–1.07) |
| 5–29 (Ref) | 1.00 | 1.00 |
| 30–49 | 0.98 (0.82–1.18) | 0.94 (0.78–1.13) |
| 50–99 | 0.81 (0.67–0.97) | **0.81 (0.67–0.97)*** |
| 100–299 | 0.79 (0.67–0.93) | 0.91 (0.78–1.08) |
| 300–999 | 0.67 (0.55–0.81) | 0.90 (0.75–1.10) |
| ≥1000 | 0.66 (0.57–0.76) | 1.00 (0.86–1.16) |
| **Pulmonary heart disease (I26–I28)** | | |
| 1 | 1.33 (0.80–2.23) | 1.21 (0.72–2.02) |
| 2–4 | 0.98 (0.81–1.18) | 0.97 (0.80–1.17) |
| 5–29 (Ref) | 1.00 | 1.00 |
| 30–49 | 1.02 (0.82–1.26) | 1.00 (0.81–1.24) |
| 50–99 | 1.01 (0.83–1.23) | 1.02 (0.83–1.24) |
| 100–299 | 0.98 (0.82–1.17) | 1.03 (0.86–1.23) |
| 300–999 | 1.01 (0.83–1.22) | 1.11 (0.91–1.34) |
| ≥1000 | 0.91 (0.78–1.06) | 1.04 (0.89–1.21) |
| **Diseases of arteries (I70–I79)** | | |
| 1 | 1.27 (1.10–1.46) | 1.03 (0.90–1.19) |
| 2–4 | 1.08 (1.03–1.13) | **1.06 (1.01–1.11)*** |
| 5–29 (Ref) | 1.00 | 1.00 |
| 30–49 | 0.99 (0.93–1.04) | 0.94 (0.89–1.00) |
| 50–99 | 0.91 (0.86–0.96) | **0.90 (0.85–0.95)*** |
| 100–299 | 0.87 (0.83–0.91) | 0.97 (0.93–1.02) |
| 300–999 | 0.76 (0.72–0.81) | 0.97 (0.91–1.02) |
| ≥1000 | 0.68 (0.65–0.71) | 0.97 (0.93–1.02) |
| **Diseases of veins/lymphatic (I80–I89)** | | |
| 1 | 0.85 (0.75–0.97) | **0.83 (0.73–0.95)*** |
| 2–4 | 1.10 (1.06–1.14) | **1.07 (1.03–1.10)*** |
| 5–29 (Ref) | 1.00 | 1.00 |
| 30–49 | 1.01 (0.97–1.05) | 1.01 (0.97–1.06) |
| 50–99 | 0.99 (0.95–1.03) | 1.02 (0.98–1.06) |
| 100–299 | 0.90 (0.87–0.93) | 1.01 (0.97–1.05) |
| 300–999 | 0.87 (0.83–0.90) | **1.05 (1.01–1.09)*** |
| ≥1000 | 0.84 (0.82–0.87) | **1.06 (1.02–1.09)*** |
| **Other circulatory disorders (I95–I99)** | | |
| 1 | 1.56 (1.02–2.39) | 1.40 (0.91–2.14) |
| 2–4 | 1.18 (1.01–1.38) | 1.16 (0.99–1.36) |
| 5–29 (Ref) | 1.00 | 1.00 |
| 30–49 | 1.13 (0.94–1.36) | 1.11 (0.92–1.33) |
| 50–99 | 0.97 (0.81–1.16) | 0.98 (0.81–1.17) |
| 100–299 | 0.82 (0.69–0.97) | 0.89 (0.75–1.06) |
| 300–999 | 0.92 (0.77–1.09) | 1.09 (0.91–1.30) |
| ≥1000 | 0.80 (0.69–0.92) | 0.99 (0.86–1.15) |

Crude OR: unadjusted odds ratio calculated from prevalence data.

Adjusted OR: adjusted for sex and age (5-year groups).

Reference category: workplace size 5–29 workers.

*Statistically significant (95% CI excludes 1.00).

**Fig. S2** Crude and age- and sex-standardized prevalence of other cardiovascular diseases by workplace size

Open circles indicate crude prevalence; filled circles indicate standardized prevalence.

Error bars represent 95% confidence intervals.

Rates per 100000 workers.

**Fig. S3** Adjusted odds ratios with 95% confidence intervals for other cardiovascular diseases by workplace size

Reference category: workplace size 5–29 workers.

Adjusted for sex and age (5-year groups).

Red indicates statistically significant increased risk (OR > 1); blue indicates statistically significant decreased risk (OR < 1); grey indicates non-significant.

**Table S3** Sensitivity analysis: prevalence of cardiovascular diseases by workplace size, IPD ≥1 day (per 100000)

| **Disease** | **1** | **2–4** | **5–29** | **30–49** | **50–99** | **100–299** | **300–999** | **≥1000** | **Total** |
| --- | --- | --- | --- | --- | --- | --- | --- | --- | --- |
| **Hypertensive diseases (I10–I15)** | | | | | | | | | |
| Crude rate (95% CI) | 51.6 (36.7–66.5) | 45.7 (41.9–49.6) | 45.6 (43.4–47.9) | 49.6 (44.8–54.4) | 41.9 (37.8–46.0) | 38.0 (34.8–41.3) | 28.5 (25.4–31.6) | 25.5 (23.4–27.6) | 39.0 (37.8–40.1) |
| Std. rate (95% CI) | 42.2 (29.1–55.4) | 43.7 (39.9–47.4) | 43.8 (41.6–46.0) | 45.1 (40.6–49.5) | 39.2 (35.3–43.1) | 38.1 (34.8–41.4) | 31.3 (27.8–34.9) | 31.5 (28.6–34.3) | 39.1 (37.9–40.3) |
| **Ischemic heart diseases (I20–I25)** | | | | | | | | | |
| Crude rate (95% CI) | 284.7 (249.7–319.7) | 194.7 (186.8–202.7) | 190.1 (185.5–194.7) | 180.3 (171.2–189.5) | 186.7 (178.0–195.3) | 171.9 (165.0–178.9) | 155.6 (148.2–163.0) | 144.3 (139.4–149.3) | 175.5 (173.1–178.0) |
| Std. rate (95% CI) | 196.9 (171.1–222.7) | 187.1 (179.4–194.8) | 179.4 (175.0–183.8) | 166.5 (158.0–175.0) | 177.8 (169.6–186.1) | 171.9 (165.0–178.9) | 167.6 (159.6–175.6) | 165.0 (158.8–171.3) | 174.3 (171.8–176.8) |
| **Other forms of heart disease (I30–I52)** | | | | | | | | | |
| Crude rate (95% CI) | 94.1 (74.0–114.3) | 87.7 (82.4–93.1) | 87.2 (84.1–90.3) | 82.3 (76.1–88.4) | 82.2 (76.5–87.9) | 82.8 (77.9–87.6) | 79.7 (74.4–85.0) | 74.2 (70.6–77.7) | 82.6 (80.9–84.3) |
| Std. rate (95% CI) | 80.0 (61.3–98.7) | 85.4 (80.1–90.6) | 85.3 (82.2–88.4) | 78.6 (72.6–84.6) | 79.3 (73.7–84.9) | 82.7 (77.8–87.5) | 83.7 (78.1–89.4) | 80.2 (76.1–84.4) | 82.7 (81.0–84.5) |
| **Cerebrovascular diseases (I60–I69)** | | | | | | | | | |
| Crude rate (95% CI) | 256.6 (223.4–289.9) | 182.0 (174.3–189.7) | 168.4 (164.1–172.8) | 168.7 (159.9–177.6) | 163.4 (155.4–171.5) | 147.3 (140.8–153.7) | 127.9 (121.3–134.6) | 118.8 (114.3–123.3) | 153.6 (151.3–155.9) |
| Std. rate (95% CI) | 196.4 (169.4–223.4) | 167.3 (160.1–174.4) | 156.8 (152.7–160.9) | 151.5 (143.4–159.5) | 152.1 (144.4–159.7) | 150.3 (143.7–156.9) | 146.6 (138.8–154.4) | 146.7 (140.7–152.8) | 153.6 (151.2–156.0) |
| **Acute rheumatic fever (I00–I02)** | | | | | | | | | |
| Crude rate (95% CI) | 0.0 (0.0–0.0) | 0.1 (0.0–0.3) | 0.0 (0.0–0.1) | 0.0 (0.0–0.0) | 0.0 (0.0–0.0) | 0.1 (0.0–0.2) | 0.0 (0.0–0.0) | 0.0 (0.0–0.0) | 0.0 (0.0–0.1) |
| Std. rate (95% CI) | 0.0 (0.0–0.0) | 0.1 (-0.1–0.2) | 0.0 (-0.0–0.1) | 0.0 (0.0–0.0) | 0.0 (0.0–0.0) | 0.1 (-0.1–0.2) | 0.0 (0.0–0.0) | 0.0 (0.0–0.0) | 0.0 (-0.0–0.0) |
| **Chronic rheumatic heart diseases (I05–I09)** | | | | | | | | | |
| Crude rate (95% CI) | 3.4 (0.0–7.2) | 2.0 (1.2–2.8) | 1.8 (1.3–2.2) | 1.9 (1.0–2.9) | 1.4 (0.6–2.1) | 1.5 (0.8–2.1) | 0.9 (0.3–1.5) | 1.2 (0.7–1.7) | 1.5 (1.3–1.8) |
| Std. rate (95% CI) | 2.7 (-0.4–5.9) | 1.8 (1.1–2.6) | 1.6 (1.2–2.0) | 1.8 (0.9–2.8) | 1.1 (0.5–1.8) | 1.6 (0.9–2.3) | 1.1 (0.4–1.7) | 1.7 (1.0–2.4) | 1.6 (1.3–1.8) |
| **Pulmonary heart disease (I26–I28)** | | | | | | | | | |
| Crude rate (95% CI) | 9.0 (2.8–15.2) | 4.6 (3.3–5.8) | 4.6 (3.8–5.3) | 4.7 (3.2–6.2) | 4.9 (3.5–6.3) | 3.2 (2.3–4.2) | 4.6 (3.3–5.9) | 4.4 (3.5–5.3) | 4.4 (4.1–4.8) |
| Std. rate (95% CI) | 8.0 (2.1–13.9) | 4.2 (3.1–5.4) | 4.4 (3.7–5.2) | 4.7 (3.2–6.1) | 4.8 (3.4–6.1) | 3.3 (2.3–4.2) | 5.1 (3.6–6.5) | 4.5 (3.6–5.5) | 4.4 (4.0–4.8) |
| **Diseases of arteries (I70–I79)** | | | | | | | | | |
| Crude rate (95% CI) | 23.5 (13.5–33.6) | 15.8 (13.5–18.1) | 15.6 (14.3–16.9) | 13.9 (11.3–16.4) | 16.1 (13.6–18.7) | 15.7 (13.6–17.8) | 11.2 (9.2–13.2) | 12.0 (10.6–13.5) | 14.5 (13.8–15.2) |
| Std. rate (95% CI) | 17.9 (9.6–26.2) | 15.6 (13.3–17.9) | 14.8 (13.5–16.1) | 13.2 (10.8–15.7) | 15.3 (12.9–17.8) | 15.8 (13.7–17.9) | 11.9 (9.7–14.0) | 13.7 (11.9–15.4) | 14.4 (13.7–15.2) |
| **Diseases of veins/lymphatic (I80–I89)** | | | | | | | | | |
| Crude rate (95% CI) | 132.2 (108.4–156.1) | 149.3 (142.3–156.2) | 146.1 (142.1–150.2) | 162.0 (153.3–170.7) | 154.9 (147.0–162.8) | 144.1 (137.8–150.5) | 133.7 (126.9–140.5) | 130.9 (126.2–135.6) | 143.7 (141.5–146.0) |
| Std. rate (95% CI) | 112.0 (90.0–134.0) | 137.1 (130.5–143.6) | 138.9 (135.0–142.8) | 150.2 (142.0–158.4) | 146.7 (139.1–154.2) | 147.6 (141.1–154.2) | 148.9 (141.1–156.7) | 150.3 (144.4–156.2) | 144.3 (142.0–146.6) |
| **Other circulatory disorders (I95–I99)** | | | | | | | | | |
| Crude rate (95% CI) | 12.3 (5.0–19.6) | 10.5 (8.6–12.3) | 10.1 (9.1–11.2) | 9.2 (7.1–11.2) | 10.1 (8.1–12.1) | 9.4 (7.8–11.0) | 10.1 (8.3–12.0) | 18.6 (16.8–20.4) | 11.7 (11.1–12.4) |
| Std. rate (95% CI) | 11.0 (4.0–18.0) | 10.2 (8.3–12.0) | 9.8 (8.8–10.8) | 9.0 (7.0–11.1) | 10.0 (8.0–12.1) | 9.3 (7.7–11.0) | 10.3 (8.3–12.3) | 17.1 (15.4–18.8) | 11.3 (10.6–11.9) |

Rates per 100000 workers.

Crude rate: Wald 95% CI.

Std. rate: age- and sex-standardized to the total study population.

Abbreviations: CI, confidence interval; IPD, inpatient days; Std., standardized.

**Table S4** Sensitivity analysis: prevalence of cardiovascular diseases by workplace size, IPD ≥3 days (per 100000)

| **Disease** | **1** | **2–4** | **5–29** | **30–49** | **50–99** | **100–299** | **300–999** | **≥1000** | **Total** |
| --- | --- | --- | --- | --- | --- | --- | --- | --- | --- |
| **Hypertensive diseases (I10–I15)** | | | | | | | | | |
| Crude rate (95% CI) | 41.5 (28.1–54.8) | 39.3 (35.7–42.9) | 39.9 (37.8–42.0) | 43.4 (38.9–47.9) | 36.6 (32.7–40.4) | 33.2 (30.2–36.3) | 23.9 (21.0–26.8) | 21.9 (20.0–23.9) | 33.8 (32.7–34.9) |
| Std. rate (95% CI) | 35.0 (22.7–47.2) | 37.6 (34.1–41.1) | 38.4 (36.4–40.5) | 39.9 (35.7–44.1) | 34.4 (30.8–38.1) | 33.2 (30.2–36.3) | 26.1 (22.9–29.3) | 27.0 (24.4–29.7) | 33.9 (32.8–35.1) |
| **Ischemic heart diseases (I20–I25)** | | | | | | | | | |
| Crude rate (95% CI) | 248.8 (216.1–281.5) | 168.5 (161.1–175.9) | 166.1 (161.7–170.4) | 157.9 (149.3–166.4) | 162.9 (154.8–171.0) | 148.7 (142.3–155.2) | 133.3 (126.5–140.2) | 121.7 (117.1–126.2) | 151.8 (149.6–154.1) |
| Std. rate (95% CI) | 170.8 (146.9–194.6) | 162.1 (154.9–169.2) | 156.4 (152.3–160.5) | 145.7 (137.7–153.6) | 155.2 (147.4–162.9) | 148.9 (142.5–155.4) | 144.3 (136.8–151.7) | 141.0 (135.2–146.8) | 151.0 (148.7–153.4) |
| **Other forms of heart disease (I30–I52)** | | | | | | | | | |
| Crude rate (95% CI) | 74.0 (56.1–91.8) | 72.2 (67.3–77.0) | 69.1 (66.4–71.9) | 67.1 (61.5–72.6) | 65.8 (60.7–71.0) | 67.6 (63.2–71.9) | 60.6 (56.0–65.2) | 56.0 (52.9–59.1) | 65.4 (63.9–66.9) |
| Std. rate (95% CI) | 60.3 (44.5–76.0) | 70.2 (65.4–75.0) | 67.5 (64.8–70.3) | 63.8 (58.4–69.2) | 63.1 (58.1–68.1) | 67.3 (63.0–71.7) | 64.2 (59.2–69.1) | 61.8 (58.1–65.5) | 65.6 (64.0–67.1) |
| **Cerebrovascular diseases (I60–I69)** | | | | | | | | | |
| Crude rate (95% CI) | 228.6 (197.3–260.0) | 162.1 (154.9–169.4) | 152.0 (147.8–156.1) | 150.2 (141.8–158.5) | 147.3 (139.6–155.0) | 130.6 (124.6–136.7) | 111.6 (105.4–117.9) | 100.9 (96.7–105.0) | 136.3 (134.1–138.4) |
| Std. rate (95% CI) | 173.8 (148.5–199.2) | 149.6 (142.9–156.4) | 141.6 (137.7–145.4) | 135.0 (127.3–142.6) | 136.8 (129.6–144.0) | 133.1 (127.0–139.3) | 127.9 (120.6–135.1) | 125.7 (120.1–131.4) | 136.2 (134.0–138.5) |
| **Acute rheumatic fever (I00–I02)** | | | | | | | | | |
| Crude rate (95% CI) | 0.0 (0.0–0.0) | 0.1 (0.0–0.3) | 0.0 (0.0–0.1) | 0.0 (0.0–0.0) | 0.0 (0.0–0.0) | 0.0 (0.0–0.0) | 0.0 (0.0–0.0) | 0.0 (0.0–0.0) | 0.0 (0.0–0.0) |
| Std. rate (95% CI) | 0.0 (0.0–0.0) | 0.1 (-0.1–0.2) | 0.0 (-0.0–0.1) | 0.0 (0.0–0.0) | 0.0 (0.0–0.0) | 0.0 (0.0–0.0) | 0.0 (0.0–0.0) | 0.0 (0.0–0.0) | 0.0 (-0.0–0.0) |
| **Chronic rheumatic heart diseases (I05–I09)** | | | | | | | | | |
| Crude rate (95% CI) | 3.4 (0.0–7.2) | 1.9 (1.1–2.6) | 1.6 (1.2–2.1) | 1.8 (0.9–2.7) | 1.3 (0.5–2.0) | 1.5 (0.8–2.1) | 0.9 (0.3–1.5) | 1.1 (0.7–1.5) | 1.5 (1.2–1.7) |
| Std. rate (95% CI) | 2.7 (-0.4–5.9) | 1.7 (1.0–2.4) | 1.5 (1.1–1.8) | 1.7 (0.8–2.6) | 1.0 (0.4–1.6) | 1.6 (0.9–2.3) | 1.1 (0.4–1.7) | 1.6 (0.9–2.3) | 1.5 (1.2–1.7) |
| **Pulmonary heart disease (I26–I28)** | | | | | | | | | |
| Crude rate (95% CI) | 9.0 (2.8–15.2) | 4.3 (3.1–5.5) | 4.5 (3.8–5.2) | 4.1 (2.7–5.5) | 4.6 (3.2–5.9) | 3.1 (2.2–4.1) | 4.5 (3.3–5.8) | 4.2 (3.4–5.1) | 4.3 (3.9–4.6) |
| Std. rate (95% CI) | 8.0 (2.1–13.9) | 4.0 (2.9–5.1) | 4.4 (3.7–5.1) | 4.0 (2.6–5.4) | 4.4 (3.1–5.8) | 3.2 (2.2–4.2) | 4.9 (3.5–6.3) | 4.4 (3.4–5.3) | 4.2 (3.9–4.6) |
| **Diseases of arteries (I70–I79)** | | | | | | | | | |
| Crude rate (95% CI) | 23.5 (13.5–33.6) | 14.5 (12.3–16.6) | 14.6 (13.3–15.9) | 12.3 (9.9–14.7) | 15.5 (13.0–18.0) | 14.5 (12.5–16.5) | 10.2 (8.3–12.1) | 11.1 (9.7–12.5) | 13.4 (12.7–14.1) |
| Std. rate (95% CI) | 17.9 (9.6–26.2) | 14.2 (12.0–16.4) | 13.8 (12.6–15.0) | 11.7 (9.4–14.0) | 14.7 (12.4–17.1) | 14.6 (12.6–16.7) | 10.8 (8.8–12.9) | 12.6 (10.9–14.3) | 13.4 (12.7–14.1) |
| **Diseases of veins/lymphatic (I80–I89)** | | | | | | | | | |
| Crude rate (95% CI) | 96.4 (76.0–116.8) | 110.1 (104.1–116.1) | 106.3 (102.8–109.7) | 116.8 (109.4–124.1) | 112.5 (105.8–119.2) | 104.9 (99.4–110.3) | 93.9 (88.2–99.6) | 92.7 (88.7–96.6) | 103.8 (101.9–105.7) |
| Std. rate (95% CI) | 83.4 (64.1–102.7) | 101.4 (95.8–107.1) | 101.1 (97.7–104.4) | 108.2 (101.2–115.1) | 106.3 (99.9–112.8) | 107.4 (101.8–113.0) | 104.8 (98.3–111.4) | 107.1 (102.1–112.1) | 104.3 (102.3–106.3) |
| **Other circulatory disorders (I95–I99)** | | | | | | | | | |
| Crude rate (95% CI) | 2.2 (0.0–5.3) | 6.4 (5.0–7.9) | 5.3 (4.5–6.0) | 4.6 (3.1–6.0) | 5.6 (4.1–7.1) | 3.8 (2.8–4.8) | 4.7 (3.4–6.0) | 5.9 (4.9–6.9) | 5.2 (4.8–5.6) |
| Std. rate (95% CI) | 1.8 (-0.8–4.3) | 6.2 (4.8–7.6) | 5.1 (4.4–5.9) | 4.5 (3.0–5.9) | 5.7 (4.1–7.2) | 3.8 (2.8–4.8) | 5.0 (3.6–6.4) | 5.7 (4.7–6.7) | 5.1 (4.7–5.6) |

Rates per 100000 workers.

Crude rate: Wald 95% CI.

Std. rate: age- and sex-standardized to the total study population.

Abbreviations: CI, confidence interval; IPD, inpatient days; Std., standardized.

**Table S5** Sensitivity analysis: prevalence of cardiovascular diseases by workplace size, IPD ≥5 days (per 100000)

| **Disease** | **1** | **2–4** | **5–29** | **30–49** | **50–99** | **100–299** | **300–999** | **≥1000** | **Total** |
| --- | --- | --- | --- | --- | --- | --- | --- | --- | --- |
| **Hypertensive diseases (I10–I15)** | | | | | | | | | |
| Crude rate (95% CI) | 39.2 (26.2–52.2) | 35.8 (32.4–39.2) | 36.5 (34.5–38.5) | 40.5 (36.2–44.9) | 33.6 (30.0–37.3) | 30.5 (27.6–33.4) | 22.1 (19.3–24.8) | 19.6 (17.8–21.4) | 31.0 (29.9–32.0) |
| Std. rate (95% CI) | 33.1 (21.1–45.0) | 34.4 (31.1–37.8) | 35.1 (33.2–37.1) | 37.2 (33.1–41.2) | 31.7 (28.2–35.2) | 30.6 (27.6–33.5) | 24.1 (21.0–27.2) | 24.2 (21.7–26.7) | 31.1 (30.0–32.1) |
| **Ischemic heart diseases (I20–I25)** | | | | | | | | | |
| Crude rate (95% CI) | 244.3 (211.9–276.8) | 158.3 (151.1–165.5) | 155.5 (151.3–159.7) | 147.8 (139.5–156.0) | 153.6 (145.8–161.5) | 138.9 (132.7–145.2) | 123.9 (117.4–130.5) | 112.1 (107.7–116.5) | 141.9 (139.7–144.1) |
| Std. rate (95% CI) | 167.2 (143.6–190.8) | 152.1 (145.2–159.1) | 146.3 (142.3–150.2) | 136.2 (128.5–143.9) | 146.2 (138.7–153.7) | 139.2 (132.9–145.5) | 134.2 (127.0–141.4) | 131.2 (125.6–136.9) | 141.2 (139.0–143.5) |
| **Other forms of heart disease (I30–I52)** | | | | | | | | | |
| Crude rate (95% CI) | 63.9 (47.3–80.5) | 64.1 (59.6–68.7) | 60.5 (57.9–63.1) | 57.8 (52.6–63.0) | 57.5 (52.7–62.3) | 58.8 (54.8–62.9) | 52.3 (48.0–56.6) | 47.9 (45.1–50.8) | 56.9 (55.5–58.3) |
| Std. rate (95% CI) | 50.8 (36.6–65.0) | 62.4 (57.9–67.0) | 58.9 (56.3–61.5) | 54.8 (49.8–59.7) | 54.9 (50.3–59.5) | 58.5 (54.4–62.5) | 55.5 (50.9–60.1) | 53.4 (49.9–56.9) | 57.1 (55.6–58.5) |
| **Cerebrovascular diseases (I60–I69)** | | | | | | | | | |
| Crude rate (95% CI) | 217.4 (186.8–248.0) | 153.0 (145.9–160.0) | 141.8 (137.8–145.8) | 139.0 (130.9–147.0) | 136.6 (129.2–144.0) | 121.4 (115.5–127.2) | 102.7 (96.7–108.7) | 91.6 (87.7–95.6) | 126.5 (124.4–128.6) |
| Std. rate (95% CI) | 163.6 (139.1–188.1) | 141.3 (134.7–147.9) | 132.2 (128.4–135.9) | 124.8 (117.5–132.2) | 126.9 (120.0–133.9) | 123.4 (117.4–129.3) | 117.7 (110.7–124.7) | 115.1 (109.7–120.5) | 126.5 (124.3–128.6) |
| **Acute rheumatic fever (I00–I02)** | | | | | | | | | |
| Crude rate (95% CI) | 0.0 (0.0–0.0) | 0.1 (0.0–0.3) | 0.0 (0.0–0.1) | 0.0 (0.0–0.0) | 0.0 (0.0–0.0) | 0.0 (0.0–0.0) | 0.0 (0.0–0.0) | 0.0 (0.0–0.0) | 0.0 (0.0–0.0) |
| Std. rate (95% CI) | 0.0 (0.0–0.0) | 0.1 (-0.1–0.2) | 0.0 (-0.0–0.1) | 0.0 (0.0–0.0) | 0.0 (0.0–0.0) | 0.0 (0.0–0.0) | 0.0 (0.0–0.0) | 0.0 (0.0–0.0) | 0.0 (-0.0–0.0) |
| **Chronic rheumatic heart diseases (I05–I09)** | | | | | | | | | |
| Crude rate (95% CI) | 3.4 (0.0–7.2) | 1.9 (1.1–2.6) | 1.5 (1.1–2.0) | 1.6 (0.7–2.4) | 1.3 (0.5–2.0) | 1.4 (0.8–2.0) | 0.8 (0.3–1.3) | 1.1 (0.6–1.5) | 1.4 (1.2–1.6) |
| Std. rate (95% CI) | 2.7 (-0.4–5.9) | 1.7 (1.0–2.4) | 1.4 (1.0–1.8) | 1.5 (0.7–2.3) | 1.0 (0.4–1.6) | 1.5 (0.8–2.1) | 0.9 (0.3–1.5) | 1.6 (0.9–2.2) | 1.4 (1.2–1.6) |
| **Pulmonary heart disease (I26–I28)** | | | | | | | | | |
| Crude rate (95% CI) | 9.0 (2.8–15.2) | 4.1 (2.9–5.2) | 4.1 (3.4–4.8) | 4.0 (2.6–5.3) | 4.5 (3.1–5.8) | 3.1 (2.1–4.0) | 4.3 (3.1–5.6) | 4.0 (3.2–4.8) | 4.0 (3.7–4.4) |
| Std. rate (95% CI) | 8.0 (2.1–13.9) | 3.8 (2.7–4.9) | 4.0 (3.4–4.7) | 3.9 (2.5–5.2) | 4.3 (3.0–5.6) | 3.1 (2.2–4.1) | 4.7 (3.3–6.1) | 4.1 (3.2–5.0) | 4.0 (3.6–4.4) |
| **Diseases of arteries (I70–I79)** | | | | | | | | | |
| Crude rate (95% CI) | 21.3 (11.7–30.9) | 13.4 (11.3–15.4) | 13.6 (12.3–14.8) | 11.7 (9.4–14.0) | 14.9 (12.5–17.3) | 13.7 (11.8–15.7) | 9.9 (8.0–11.7) | 10.2 (8.9–11.5) | 12.6 (11.9–13.2) |
| Std. rate (95% CI) | 16.5 (8.5–24.6) | 13.2 (11.1–15.3) | 12.8 (11.7–14.0) | 11.1 (8.9–13.3) | 14.2 (11.8–16.5) | 13.8 (11.9–15.8) | 10.5 (8.5–12.5) | 11.7 (10.0–13.3) | 12.5 (11.9–13.2) |
| **Diseases of veins/lymphatic (I80–I89)** | | | | | | | | | |
| Crude rate (95% CI) | 76.2 (58.1–94.3) | 88.4 (83.0–93.8) | 84.1 (81.0–87.1) | 94.0 (87.4–100.6) | 89.4 (83.4–95.4) | 83.9 (79.1–88.8) | 77.2 (72.0–82.3) | 75.6 (72.0–79.2) | 83.2 (81.6–84.9) |
| Std. rate (95% CI) | 64.5 (47.8–81.3) | 81.8 (76.7–86.9) | 80.2 (77.2–83.2) | 87.0 (80.8–93.2) | 84.5 (78.8–90.3) | 85.8 (80.8–90.7) | 86.2 (80.2–92.1) | 87.1 (82.6–91.6) | 83.8 (82.0–85.5) |
| **Other circulatory disorders (I95–I99)** | | | | | | | | | |
| Crude rate (95% CI) | 1.1 (0.0–3.3) | 3.6 (2.5–4.7) | 3.5 (2.9–4.2) | 3.5 (2.2–4.8) | 3.5 (2.4–4.7) | 2.6 (1.7–3.4) | 3.0 (2.0–4.0) | 2.5 (1.9–3.2) | 3.1 (2.8–3.5) |
| Std. rate (95% CI) | 0.7 (-0.6–2.0) | 3.5 (2.5–4.6) | 3.4 (2.8–4.0) | 3.3 (2.1–4.5) | 3.6 (2.4–4.8) | 2.6 (1.7–3.4) | 3.2 (2.1–4.3) | 2.6 (1.9–3.3) | 3.1 (2.8–3.4) |

Rates per 100000 workers.

Crude rate: Wald 95% CI.

Std. rate: age- and sex-standardized to the total study population.

Abbreviations: CI, confidence interval; IPD, inpatient days; Std., standardized.

**Table S6** Sensitivity analysis: crude odds ratios for cardiovascular diseases by workplace size, IPD ≥1 day

| **Disease / Workplace size** | **Crude OR (95% CI)** |
| --- | --- |
| **Hypertensive diseases (I10–I15)** | |
| 1 | 1.13 (0.84–1.51) |
| 2–4 | 1.00 (0.91–1.10) |
| 5–29 (Ref) | 1.00 |
| 30–49 | 1.09 (0.97–1.21) |
| 50–99 | 0.92 (0.82–1.02) |
| 100–299 | **0.83 (0.75–0.92)*** |
| 300–999 | **0.62 (0.55–0.70)*** |
| ≥1000 | **0.56 (0.51–0.61)*** |
| **Ischemic heart diseases (I20–I25)** | |
| 1 | **1.50 (1.32–1.70)*** |
| 2–4 | 1.02 (0.98–1.07) |
| 5–29 (Ref) | 1.00 |
| 30–49 | 0.95 (0.90–1.00) |
| 50–99 | 0.98 (0.93–1.03) |
| 100–299 | **0.90 (0.86–0.95)*** |
| 300–999 | **0.82 (0.78–0.86)*** |
| ≥1000 | **0.76 (0.73–0.79)*** |
| **Other forms of heart disease (I30–I52)** | |
| 1 | 1.08 (0.87–1.34) |
| 2–4 | 1.01 (0.94–1.08) |
| 5–29 (Ref) | 1.00 |
| 30–49 | 0.94 (0.87–1.03) |
| 50–99 | 0.94 (0.87–1.02) |
| 100–299 | 0.95 (0.89–1.02) |
| 300–999 | **0.91 (0.85–0.99)*** |
| ≥1000 | **0.85 (0.80–0.90)*** |
| **Cerebrovascular diseases (I60–I69)** | |
| 1 | **1.53 (1.34–1.74)*** |
| 2–4 | **1.08 (1.03–1.14)*** |
| 5–29 (Ref) | 1.00 |
| 30–49 | 1.00 (0.95–1.06) |
| 50–99 | 0.97 (0.92–1.03) |
| 100–299 | **0.87 (0.83–0.92)*** |
| 300–999 | **0.76 (0.72–0.80)*** |
| ≥1000 | **0.71 (0.67–0.74)*** |
| **Acute rheumatic fever (I00–I02)** | |
| 1 | — |
| 2–4 | 2.89 (0.18–46.24) |
| 5–29 (Ref) | 1.00 |
| 30–49 | — |
| 50–99 | — |
| 100–299 | 2.50 (0.16–39.93) |
| 300–999 | — |
| ≥1000 | — |
| **Chronic rheumatic heart diseases (I05–I09)** | |
| 1 | 1.92 (0.60–6.11) |
| 2–4 | 1.16 (0.72–1.86) |
| 5–29 (Ref) | 1.00 |
| 30–49 | 1.10 (0.63–1.91) |
| 50–99 | 0.77 (0.42–1.41) |
| 100–299 | 0.83 (0.50–1.38) |
| 300–999 | 0.52 (0.26–1.01) |
| ≥1000 | 0.68 (0.43–1.08) |
| **Pulmonary heart disease (I26–I28)** | |
| 1 | 1.97 (0.97–4.00) |
| 2–4 | 1.00 (0.73–1.36) |
| 5–29 (Ref) | 1.00 |
| 30–49 | 1.03 (0.73–1.47) |
| 50–99 | 1.07 (0.78–1.49) |
| 100–299 | **0.70 (0.50–0.98)*** |
| 300–999 | 1.01 (0.74–1.39) |
| ≥1000 | 0.96 (0.75–1.24) |
| **Diseases of arteries (I70–I79)** | |
| 1 | 1.51 (0.98–2.33) |
| 2–4 | 1.01 (0.86–1.20) |
| 5–29 (Ref) | 1.00 |
| 30–49 | 0.89 (0.73–1.09) |
| 50–99 | 1.03 (0.87–1.24) |
| 100–299 | 1.01 (0.86–1.18) |
| 300–999 | **0.72 (0.59–0.87)*** |
| ≥1000 | **0.77 (0.67–0.89)*** |
| **Diseases of veins/lymphatic (I80–I89)** | |
| 1 | 0.91 (0.75–1.09) |
| 2–4 | 1.02 (0.97–1.08) |
| 5–29 (Ref) | 1.00 |
| 30–49 | **1.11 (1.04–1.18)*** |
| 50–99 | 1.06 (1.00–1.12) |
| 100–299 | 0.99 (0.94–1.04) |
| 300–999 | **0.92 (0.86–0.97)*** |
| ≥1000 | **0.90 (0.86–0.94)*** |
| **Other circulatory disorders (I95–I99)** | |
| 1 | 1.22 (0.67–2.22) |
| 2–4 | 1.03 (0.84–1.27) |
| 5–29 (Ref) | 1.00 |
| 30–49 | 0.90 (0.71–1.16) |
| 50–99 | 1.00 (0.80–1.25) |
| 100–299 | 0.93 (0.76–1.14) |
| 300–999 | 1.00 (0.81–1.24) |
| ≥1000 | **1.83 (1.59–2.11)*** |

Crude OR: unadjusted odds ratio calculated from prevalence data.

Reference category: workplace size 5–29 workers.

*Statistically significant (95% CI excludes 1.00).

Abbreviations: CI, confidence interval; IPD, inpatient days; OR, odds ratio.

**Table S7** Sensitivity analysis: crude odds ratios for cardiovascular diseases by workplace size, IPD ≥3 days

| **Disease / Workplace size** | **Crude OR (95% CI)** |
| --- | --- |
| **Hypertensive diseases (I10–I15)** | |
| 1 | 1.04 (0.75–1.44) |
| 2–4 | 0.98 (0.89–1.09) |
| 5–29 (Ref) | 1.00 |
| 30–49 | 1.09 (0.97–1.22) |
| 50–99 | 0.92 (0.81–1.03) |
| 100–299 | **0.83 (0.75–0.92)*** |
| 300–999 | **0.60 (0.52–0.68)*** |
| ≥1000 | **0.55 (0.50–0.61)*** |
| **Ischemic heart diseases (I20–I25)** | |
| 1 | **1.50 (1.31–1.71)*** |
| 2–4 | 1.01 (0.96–1.07) |
| 5–29 (Ref) | 1.00 |
| 30–49 | 0.95 (0.90–1.01) |
| 50–99 | 0.98 (0.93–1.04) |
| 100–299 | **0.90 (0.85–0.94)*** |
| 300–999 | **0.80 (0.76–0.85)*** |
| ≥1000 | **0.73 (0.70–0.77)*** |
| **Other forms of heart disease (I30–I52)** | |
| 1 | 1.07 (0.84–1.37) |
| 2–4 | 1.04 (0.97–1.13) |
| 5–29 (Ref) | 1.00 |
| 30–49 | 0.97 (0.88–1.06) |
| 50–99 | 0.95 (0.87–1.04) |
| 100–299 | 0.98 (0.91–1.05) |
| 300–999 | **0.88 (0.80–0.96)*** |
| ≥1000 | **0.81 (0.76–0.87)*** |
| **Cerebrovascular diseases (I60–I69)** | |
| 1 | **1.51 (1.31–1.73)*** |
| 2–4 | **1.07 (1.01–1.12)*** |
| 5–29 (Ref) | 1.00 |
| 30–49 | 0.99 (0.93–1.05) |
| 50–99 | 0.97 (0.91–1.03) |
| 100–299 | **0.86 (0.81–0.91)*** |
| 300–999 | **0.73 (0.69–0.78)*** |
| ≥1000 | **0.66 (0.63–0.70)*** |
| **Acute rheumatic fever (I00–I02)** | |
| 1 | — |
| 2–4 | 2.89 (0.18–46.24) |
| 5–29 (Ref) | 1.00 |
| 30–49 | — |
| 50–99 | — |
| 100–299 | — |
| 300–999 | — |
| ≥1000 | — |
| **Chronic rheumatic heart diseases (I05–I09)** | |
| 1 | 2.05 (0.64–6.56) |
| 2–4 | 1.14 (0.69–1.86) |
| 5–29 (Ref) | 1.00 |
| 30–49 | 1.11 (0.63–1.95) |
| 50–99 | 0.76 (0.41–1.42) |
| 100–299 | 0.89 (0.54–1.49) |
| 300–999 | 0.55 (0.28–1.08) |
| ≥1000 | 0.68 (0.42–1.09) |
| **Pulmonary heart disease (I26–I28)** | |
| 1 | 2.01 (0.99–4.08) |
| 2–4 | 0.96 (0.70–1.32) |
| 5–29 (Ref) | 1.00 |
| 30–49 | 0.92 (0.63–1.33) |
| 50–99 | 1.03 (0.73–1.43) |
| 100–299 | **0.70 (0.50–0.98)*** |
| 300–999 | 1.01 (0.74–1.39) |
| ≥1000 | 0.94 (0.73–1.22) |
| **Diseases of arteries (I70–I79)** | |
| 1 | **1.61 (1.04–2.50)*** |
| 2–4 | 0.99 (0.83–1.18) |
| 5–29 (Ref) | 1.00 |
| 30–49 | 0.84 (0.68–1.04) |
| 50–99 | 1.06 (0.89–1.28) |
| 100–299 | 1.00 (0.84–1.17) |
| 300–999 | **0.70 (0.57–0.86)*** |
| ≥1000 | **0.76 (0.65–0.89)*** |
| **Diseases of veins/lymphatic (I80–I89)** | |
| 1 | 0.91 (0.73–1.12) |
| 2–4 | 1.04 (0.97–1.10) |
| 5–29 (Ref) | 1.00 |
| 30–49 | **1.10 (1.02–1.18)*** |
| 50–99 | 1.06 (0.99–1.13) |
| 100–299 | 0.99 (0.93–1.05) |
| 300–999 | **0.88 (0.82–0.95)*** |
| ≥1000 | **0.87 (0.83–0.92)*** |
| **Other circulatory disorders (I95–I99)** | |
| 1 | 0.43 (0.11–1.72) |
| 2–4 | 1.22 (0.93–1.60) |
| 5–29 (Ref) | 1.00 |
| 30–49 | 0.87 (0.61–1.24) |
| 50–99 | 1.07 (0.79–1.45) |
| 100–299 | **0.72 (0.53–0.98)*** |
| 300–999 | 0.89 (0.66–1.22) |
| ≥1000 | 1.11 (0.89–1.39) |

Crude OR: unadjusted odds ratio calculated from prevalence data.

Reference category: workplace size 5–29 workers.

*Statistically significant (95% CI excludes 1.00).

Abbreviations: CI, confidence interval; IPD, inpatient days; OR, odds ratio.

**Table S8** Sensitivity analysis: crude odds ratios for cardiovascular diseases by workplace size, IPD ≥5 days

| **Disease / Workplace size** | **Crude OR (95% CI)** |
| --- | --- |
| **Hypertensive diseases (I10–I15)** | |
| 1 | 1.07 (0.77–1.50) |
| 2–4 | 0.98 (0.88–1.10) |
| 5–29 (Ref) | 1.00 |
| 30–49 | 1.11 (0.98–1.25) |
| 50–99 | 0.92 (0.82–1.04) |
| 100–299 | **0.84 (0.75–0.93)*** |
| 300–999 | **0.60 (0.53–0.69)*** |
| ≥1000 | **0.54 (0.48–0.60)*** |
| **Ischemic heart diseases (I20–I25)** | |
| 1 | **1.57 (1.37–1.80)*** |
| 2–4 | 1.02 (0.97–1.07) |
| 5–29 (Ref) | 1.00 |
| 30–49 | 0.95 (0.89–1.01) |
| 50–99 | 0.99 (0.93–1.05) |
| 100–299 | **0.89 (0.85–0.94)*** |
| 300–999 | **0.80 (0.75–0.85)*** |
| ≥1000 | **0.72 (0.69–0.76)*** |
| **Other forms of heart disease (I30–I52)** | |
| 1 | 1.06 (0.81–1.37) |
| 2–4 | 1.06 (0.98–1.15) |
| 5–29 (Ref) | 1.00 |
| 30–49 | 0.96 (0.87–1.06) |
| 50–99 | 0.95 (0.87–1.04) |
| 100–299 | 0.97 (0.90–1.06) |
| 300–999 | **0.86 (0.79–0.95)*** |
| ≥1000 | **0.79 (0.74–0.85)*** |
| **Cerebrovascular diseases (I60–I69)** | |
| 1 | **1.53 (1.33–1.77)*** |
| 2–4 | **1.08 (1.02–1.14)*** |
| 5–29 (Ref) | 1.00 |
| 30–49 | 0.98 (0.92–1.05) |
| 50–99 | 0.96 (0.91–1.02) |
| 100–299 | **0.86 (0.81–0.90)*** |
| 300–999 | **0.72 (0.68–0.77)*** |
| ≥1000 | **0.65 (0.61–0.68)*** |
| **Acute rheumatic fever (I00–I02)** | |
| 1 | — |
| 2–4 | 2.89 (0.18–46.24) |
| 5–29 (Ref) | 1.00 |
| 30–49 | — |
| 50–99 | — |
| 100–299 | — |
| 300–999 | — |
| ≥1000 | — |
| **Chronic rheumatic heart diseases (I05–I09)** | |
| 1 | 2.17 (0.68–6.95) |
| 2–4 | 1.20 (0.73–1.97) |
| 5–29 (Ref) | 1.00 |
| 30–49 | 1.01 (0.55–1.86) |
| 50–99 | 0.81 (0.43–1.51) |
| 100–299 | 0.90 (0.53–1.51) |
| 300–999 | 0.53 (0.26–1.07) |
| ≥1000 | 0.69 (0.42–1.11) |
| **Pulmonary heart disease (I26–I28)** | |
| 1 | **2.18 (1.07–4.44)*** |
| 2–4 | 0.98 (0.71–1.37) |
| 5–29 (Ref) | 1.00 |
| 30–49 | 0.97 (0.66–1.41) |
| 50–99 | 1.09 (0.77–1.53) |
| 100–299 | 0.74 (0.53–1.05) |
| 300–999 | 1.05 (0.76–1.46) |
| ≥1000 | 0.97 (0.74–1.26) |
| **Diseases of arteries (I70–I79)** | |
| 1 | 1.57 (0.99–2.49) |
| 2–4 | 0.98 (0.82–1.18) |
| 5–29 (Ref) | 1.00 |
| 30–49 | 0.86 (0.69–1.07) |
| 50–99 | 1.10 (0.91–1.33) |
| 100–299 | 1.01 (0.85–1.20) |
| 300–999 | **0.73 (0.59–0.90)*** |
| ≥1000 | **0.75 (0.64–0.88)*** |
| **Diseases of veins/lymphatic (I80–I89)** | |
| 1 | 0.91 (0.71–1.15) |
| 2–4 | 1.05 (0.98–1.13) |
| 5–29 (Ref) | 1.00 |
| 30–49 | **1.12 (1.03–1.21)*** |
| 50–99 | 1.06 (0.99–1.15) |
| 100–299 | 1.00 (0.93–1.07) |
| 300–999 | **0.92 (0.85–0.99)*** |
| ≥1000 | **0.90 (0.85–0.95)*** |
| **Other circulatory disorders (I95–I99)** | |
| 1 | 0.32 (0.04–2.27) |
| 2–4 | 1.03 (0.73–1.46) |
| 5–29 (Ref) | 1.00 |
| 30–49 | 0.99 (0.66–1.48) |
| 50–99 | 1.00 (0.68–1.47) |
| 100–299 | 0.72 (0.50–1.05) |
| 300–999 | 0.84 (0.57–1.24) |
| ≥1000 | **0.72 (0.52–0.98)*** |

Crude OR: unadjusted odds ratio calculated from prevalence data.

Reference category: workplace size 5–29 workers.

*Statistically significant (95% CI excludes 1.00).

Abbreviations: CI, confidence interval; IPD, inpatient days; OR, odds ratio.

**Table S9** Adjusted odds ratios for cardiovascular disease subtypes by workplace size, adjusted for industry type

| **Disease / Workplace size** | **aOR (95% CI)ᵃ** | **aOR (95% CI)ᵇ** |
| --- | --- | --- |
| **Hypertensive diseases (I10–I15)** | | |
| 1 | **0.85 (0.83–0.87)*** | **0.85 (0.83–0.87)*** |
| 2–4 | **0.94 (0.93–0.95)*** | **0.93 (0.92–0.93)*** |
| 5–29 (Reference) | 1.00 | 1.00 |
| 30–49 | **1.07 (1.06–1.08)*** | **1.07 (1.06–1.08)*** |
| 50–99 | **1.09 (1.08–1.10)*** | **1.09 (1.08–1.10)*** |
| 100–299 | **1.06 (1.05–1.07)*** | **1.06 (1.06–1.07)*** |
| 300–999 | **1.03 (1.03–1.04)*** | **1.04 (1.03–1.05)*** |
| ≥1000 | **0.94 (0.93–0.95)*** | **0.95 (0.94–0.95)*** |
| **Ischemic heart diseases (I20–I25)** | | |
| 1 | **1.12 (1.05–1.19)*** | **1.11 (1.04–1.18)*** |
| 2–4 | **1.05 (1.03–1.08)*** | **1.06 (1.03–1.08)*** |
| 5–29 (Reference) | 1.00 | 1.00 |
| 30–49 | 0.98 (0.95–1.01) | **0.97 (0.94–1.00)*** |
| 50–99 | 0.99 (0.96–1.01) | **0.96 (0.94–0.99)*** |
| 100–299 | 0.98 (0.95–1.00) | **0.96 (0.93–0.98)*** |
| 300–999 | **0.96 (0.94–0.99)*** | **0.96 (0.93–0.99)*** |
| ≥1000 | 1.00 (0.98–1.02) | 0.98 (0.96–1.01) |
| **Other forms of heart disease (I30–I52)** | | |
| 1 | 1.03 (0.94–1.13) | 1.03 (0.94–1.12) |
| 2–4 | 1.02 (0.99–1.06) | **1.04 (1.00–1.07)*** |
| 5–29 (Reference) | 1.00 | 1.00 |
| 30–49 | 0.97 (0.94–1.01) | **0.96 (0.93–1.00)*** |
| 50–99 | 0.97 (0.94–1.01) | **0.96 (0.93–0.99)*** |
| 100–299 | 0.97 (0.94–1.00) | **0.97 (0.94–1.00)*** |
| 300–999 | **1.06 (1.02–1.09)*** | **1.05 (1.02–1.09)*** |
| ≥1000 | **1.08 (1.05–1.11)*** | **1.08 (1.05–1.11)*** |
| **Cerebrovascular diseases (I60–I69)** | | |
| 1 | 1.04 (0.97–1.12) | 1.03 (0.95–1.11) |
| 2–4 | **1.03 (1.00–1.06)*** | **1.03 (1.01–1.06)*** |
| 5–29 (Reference) | 1.00 | 1.00 |
| 30–49 | 0.99 (0.96–1.02) | 0.97 (0.94–1.00) |
| 50–99 | **0.94 (0.91–0.97)*** | **0.91 (0.89–0.94)*** |
| 100–299 | **0.97 (0.94–0.99)*** | **0.93 (0.91–0.96)*** |
| 300–999 | 0.99 (0.96–1.02) | **0.96 (0.93–0.99)*** |
| ≥1000 | **1.06 (1.03–1.08)*** | 1.01 (0.99–1.04) |

ᵃ Adjusted for sex and age (5-year groups). Reference: 5–29 workers.

ᵇ Additionally adjusted for industry type (21 categories, KSIC-based). Reference: Manufacturing.

*Statistically significant (95% CI excludes 1.00).

**Table S10** E-values for the association between workplace size and cardiovascular disease subtypes

| **Disease / Workplace size** | **aOR (95% CI)** | **E-value (point estimate)** | **E-value (confidence interval)** |
| --- | --- | --- | --- |
| **Hypertensive diseases (I10–I15)** | | | |
| 1 | **0.85 (0.83–0.87)*** | 1.58 | 1.52 |
| 2–4 | **0.94 (0.93–0.95)*** | 1.31 | 1.28 |
| 30–49 | **1.07 (1.06–1.08)*** | 1.32 | 1.29 |
| 50–99 | **1.09 (1.08–1.10)*** | 1.36 | 1.34 |
| 100–299 | **1.06 (1.05–1.07)*** | 1.29 | 1.27 |
| 300–999 | **1.03 (1.03–1.04)*** | 1.20 | 1.17 |
| ≥1000 | **0.94 (0.93–0.95)*** | 1.31 | 1.29 |
| **Ischemic heart diseases (I20–I25)** | | | |
| 1 | **1.12 (1.05–1.19)*** | 1.48 | 1.27 |
| 2–4 | **1.05 (1.03–1.08)*** | 1.29 | 1.20 |
| 30–49 | 0.98 (0.95–1.01) | 1.17 | 1.00 |
| 50–99 | 0.99 (0.96–1.01) | 1.14 | 1.00 |
| 100–299 | 0.98 (0.95–1.00) | 1.18 | 1.00 |
| 300–999 | **0.96 (0.94–0.99)*** | 1.24 | 1.10 |
| ≥1000 | 1.00 (0.98–1.02) | 1.03 | 1.00 |
| **Other forms of heart disease (I30–I52)** | | | |
| 1 | 1.03 (0.94–1.13) | 1.20 | 1.00 |
| 2–4 | 1.02 (0.99–1.06) | 1.18 | 1.00 |
| 30–49 | 0.97 (0.94–1.01) | 1.21 | 1.00 |
| 50–99 | 0.97 (0.94–1.01) | 1.19 | 1.00 |
| 100–299 | 0.97 (0.94–1.00) | 1.20 | 1.00 |
| 300–999 | **1.06 (1.02–1.09)*** | 1.31 | 1.17 |
| ≥1000 | **1.08 (1.05–1.11)*** | 1.37 | 1.28 |
| **Cerebrovascular diseases (I60–I69)** | | | |
| 1 | 1.04 (0.97–1.12) | 1.25 | 1.00 |
| 2–4 | **1.03 (1.00–1.06)*** | 1.21 | 1.07 |
| 30–49 | 0.99 (0.96–1.02) | 1.12 | 1.00 |
| 50–99 | **0.94 (0.91–0.97)*** | 1.32 | 1.21 |
| 100–299 | **0.97 (0.94–0.99)*** | 1.23 | 1.10 |
| 300–999 | 0.99 (0.96–1.02) | 1.12 | 1.00 |
| ≥1000 | **1.06 (1.03–1.08)*** | 1.30 | 1.21 |

Model adjusted for sex and age (5-year groups). Reference: 5–29 workers.

E-values were calculated using the formula: E-value = RR + √(RR × (RR − 1)), where RR ≈ aOR under the rare disease assumption (VanderWeele and Ding, 2017).

For protective associations (aOR < 1), the reciprocal of the aOR (1/aOR) was used.

E-value for the CI was calculated using the confidence limit closest to the null (1.00). When the 95% CI included 1.00, the E-value was set to 1.00.

*Statistically significant (95% CI excludes 1.00).
